# Fear learning and extinction predicts anxiety in daily life: a study of Pavlovian conditioning and ecological momentary assessment

**DOI:** 10.1017/S0033291722002379

**Published:** 2023-08

**Authors:** Kathryn L. Modecki, Katherine M. Ryan, Allison M. Waters

**Affiliations:** 1 Menzies Health Institute Queensland; Centre for Mental Health, School of Applied Psychology, Griffith University, Mt Gravatt, Queensland, Australia; 2 School of Applied Psychology Griffith University Mt Gravatt, Queensland, Australia; 3 Centre for Mental Health; School of Applied Psychology Griffith University Mt Gravatt, Queensland, Australia

**Keywords:** Ecological momentary assessment (EMA), Pavlovian conditioning, fear learning, anxiety

## Abstract

**Background:**

The association between anxious mood and aberrant fear learning mechanisms has not been fully elucidated. Studying how fear conditioning and extinction constructs relate to anxiety symptoms and reactivity to stressful and benign moments in everyday life provides a powerful addition to experimental paradigms.

**Method:**

Fifty-one young adults completed laboratory-based differential conditioning and extinction tasks with (CS + ) and without (CS-) an aversive unconditional stimulus (US). Electrodermal skin conductance responses were measured during each phase, followed by ecological momentary assessment (EMA) tapping anxiety and stressors six times daily for seven days (2, 142 moments).

**Results:**

Conditioned electrodermal reactivity to the CS + and overgeneralisation to the CS- were associated with greater change in anxiety (measured via EMA), across non-stressful situations, remaining the same across stressful situations. Likewise, during extinction when the CS + is now safe, more electrodermal reactivity to the CS + was associated with more anxiety change across non-stressful situations and remained the same across stressful situations. Also, during extinction when threat is absent, more electrodermal reactivity at the late stage of the CS- was associated with less momentary anxiety change in response to stressful situations; more electrodermal activity at the late stage of the CS + was associated with more anxiety change across non-stressful situations and remained the same across stressful situations.

**Conclusions:**

Sampling ‘in vivo’ emotion and stress experiences, study findings revealed links between conditioned electrodermal reactivity and overgeneralisation to safe stimuli and heightened anxious reactivity during non-stressful (i.e. safe) moments in daily life, coupled with less change in response to actual stressors.

With an estimated lifetime prevalence of nearly a third of individuals worldwide, anxiety disorders represent a major health and economic burden (Kessler et al., 2005; Marciniak, Lage, Landbloom, Dunayevich, & Bowman, 2004). Fear conditioning and extinction models show significant promise in explaining how anxiety develops within laboratory-based Pavlovian conditioning tasks (Lonsdorf et al., 2017) and can be treated via extinction principles underlying exposure therapy in CBT (Heinig et al., 2017).

Pavlovian fear learning models of anxiety are widely supported (Duits et al., 2015) and indicate that anxiety develops through the association of a conditioned stimulus (CS + ) and an aversive, previously benign unconditioned stimulus (US), so that the CS + , in itself, is capable of eliciting anxiety whereas a stimulus that is never paired with the US will not (CS-). Importantly, during an extinction phase, the CS + is repeatedly presented without the unconditioned stimulus so that eventually, the heighted reactivity to the CS + begins to weaken and the learned fear is extinguished. More anxious individuals exhibit elevated electrodermal (measured via skin conductance) responses to the CS + and CS- during fear conditioning and exhibit perturbations in reducing responses to the CS + when it is no longer paired with threat during extinction (i.e. overgeneralisation) (Duits et al., 2015; Dymond, Dunsmoor, Vervliet, Roche, & Hermans, 2015).

However, the association between aberrant fear learning mechanisms and anxious mood has not been fully elucidated. Theoretical tenets underlying aberrant fear learning is that more anxious individuals (a) more readily acquire elevated conditioned fear responses, (b) generalise these associations to safe stimuli, and (c) have difficulty inhibiting conditioned responses with new information indicating that the CS + is safe during extinction (Scheveneels, Boddez, & Hermans, 2021). Moreover, a major premise is that these individuals are more likely to generalise these heightened associative processes to everyday stressors and benign events which do not necessitate a ‘reactive’ response (Craske, Treanor, Conway, Zbozinek, & Vervliet, 2014).

To date, however, no studies have examined the association of Pavlovian fear conditioning, overgeneralisation to safe stimuli, and impaired extinction with anxiety symptoms in daily life. Given extant data, other related studies may be relevant which have demonstrated that elevated defensive responding, in the form of larger startle eye blink responses to safe stimuli during fear-potentiated startle eye blink experiments, characterise individuals with anxiety disorders (e.g. Grillon, Ameli, Goddard, Woods, and Davis, 1994; Waters et al., 2014) and predict anxiety disorder onset four years later (Craske *et al*. 2012). Several additional studies provide preliminary support for reduced extinction of a conditioned emotional response as a vulnerability factor for posttraumatic stress (Lommen, van de Schoot, & Engelhard, 2014; Orr et al., 2012). However, no studies have examined the translation of fear learning processes to ‘in vivo’ experiences of anxiety in everyday moments and stressors across daily life. Such research is critical for characterising whether laboratory-based measures of fear learning impairments predict everyday fluctuations in anxiety symptoms and maladjustment to stressful and benign (i.e. safe) moments in day-to-day life (Scheveneels, Boddez, Vervliet, & Hermans, 2016).

In this study, we aimed to characterise the relations between fear acquisition and extinction constructs based on electrodermal (skin conductance) measures indexed within Pavlovian fear conditioning and extinction tasks and everyday anxious reactivity to stressful and non-stressful (i.e. safe) events measured via ecological momentary assessment (EMA). EMA leverages individuals' reporting of their experiences (including stressors, positive, and benign events) and their current emotions in the moment, across various time points and settings (Myin-Germeys et al., 2018, 2009). Because each individual is their own control, EMA facilitates modelling of emotional experiences, including individuals' emotion reactivity (i.e. change in emotion) in relation to events (Hamaker, 2012; Telford, McCarthy-Jones, Corcoran, & Rowse, 2012). Thus, EMA offers excellent ecological validity for measuring stress reactivity across daily life (Modecki, Duvenage, Uink, Barber, & Donovan, 2022; Modecki, Goldberg, Ehrenreich, Russell, & Bellmore, 2019).

We hypothesised that if heightened fear conditioning and overgeneralisation to safe stimuli heightens reactivity in daily life (Duits et al., 2015; Waters et al., 2014), then larger electrodermal responses to the CS + and the safe stimulus (CS-) during conditioning will predict elevated anxiety in relation to safe situations (i.e. no stress situations) comparable to the degree of anxiety in relation to threat situations (i.e. stressors) in daily life. Predictions regarding extinction are less clear in the absence of prior studies. However, if slowed extinction and overgeneralisation relate to difficulty inhibiting anxious reactivity in daily life (Lommen et al., 2014), then elevated electrodermal responses to the CS + and CS- during extinction will predict less change in anxiety following a stressor (that is, anxiety level after a stressor, controlling for anxiety at the prior moment) by virtue of elevated anxiety reactivity across non-stressful moments.

## Methods

### Participants

Original study participants were 62 young adults (71% female; 29% male) age 17–28 years (*M_age_* = 19.11, *s.d.* = 2.77) recruited via a University research pool. Participants were awarded course credit for phase one and a gift card for phase two to the value of the amount of their SMS responses to the EMA protocol (up to $45). Participants were drawn from studies running in the lab (see Stimulus Materials). Two participants from the lab-based sessions declined to participate in the EMA phase (97% uptake); nine participants who completed the EMA phase were not in a control study arm (e.g. were exposed to an additional task prior to fear conditioning) and so were subsequently excluded. Thus, the final sample consisted of 51 young adults (*M_age_* = 18.96, *s.d.* = 2.70, 17–28 years; 70% female).

### Measures

#### Anxiety symptoms

The State-Trait Anxiety Inventory for Adults (STAI; Spielberger, Jacobs, Russell, and Crane, 1983) was used to assess anxiety symptomology. We used the 20-item trait sub-scale designed to differentiate the more general and longstanding quality of ‘trait anxiety,’ from short-term, fluctuations in state anxiety, which are better captured within our ESM design. Higher scores indicating higher symptomology/anxiety, *M* = 44.23, *s.d.* = 10.88, range = 23–67, *α* = 0.91. Six participants were not given the STAI to complete; they were still included in the models in accordance with best practice (Enders, 2022).

#### Phase one: Conditioning and extinction task

#### Stimulus materials

Two different stimuli were used across four individual studies which contributed to the current project, each of which was a fear conditioning and extinction task. Studies (#1 and #2) used geometric shapes of a pastel cream triangle and pastel pink trapezoid as the CSs. The US was a 3s recording of a three-pronged garden fork scraped over slate (see Neumann & Waters, 2006; Waters, Theresiana, Neumann, & Craske, 2017). Two studies (#3 and #4), used photographs of dogs as the CSs. The US was a 3s aversive sound of a dog growling and a woman screaming reaching 100 dB. The dichotomous study stimulus covariate was included in all models and was non-significant. See online Supplementary Section for details related to individual studies and study stimuli and additional sensitivity checks associated with each.

#### Skin conductance responses (SCR)

SCR were recorded using two self-adhesive isotonic electrodes (Biopac systems EL507) attached to the thenar and hypothenar eminences of the non-dominant hand. Data were analysed using AcqKnowledge software Version 5.0. See online Supplementary section for details and descriptives.

#### Phase two: Ecological Momentary Assessment (EMA)

#### Momentary emotion

Participants were asked *‘Please indicate how _____ you feel right now?’* at each sampling moment. Participants rated how happy, sad, angry, and anxious they were feeling (1 = Not at all, 5 = Very much) (Schneiders et al., 2006; Uink, Modecki, & Barber, 2017). For the current study, we focused on anxious emotions in relation to stress responses, *M* = 1.89, *s.d.* = 1.19.

#### Momentary stress

Participants were asked ‘Since the last set of questions, has anything negative or stressful/bad happened to you?’ at each sampling moment (Schneiders et al., 2006; Uink, Modecki, Barber, & Correia, 2018). The question format ensured that participants reported on recent stressors in the last several hours. Participants reported stressful events at approximately 12% of all sampling moments. Responses were dummy coded (0 = no bad event since last messaged, 1 = bad event since last messaged) (blinded).

#### Momentary uplifts

Participants also reported on momentary uplifts in a similar fashion ‘Since the last set of questions, has anything good or positive happened to you?’ to control for these experiences in all models (Uink, Modecki, Barber, & Correia, 2018). Participants reported uplifts at approximately 18% of all sampling moments. The online Supplementary Section incudes full details for all EMA questions.

### Procedure

Approval was obtained from the University Ethics Committee to undertake the fear conditioning tasks (# 2019/167; 2019/165; 2018/891) and to conduct a week-long EMA follow-up (#2018/499). For phase 1, participants completed the fear conditioning and extinction task in one of four studies and following the lab session, were recruited for phase two. Participants provided their phone number and returned the signed consent form via email to participate in phase two. Subsequently, participants were called/texted to assign a start date.

#### Phase 1: fear conditioning, extinction and retest

All studies involved the same number of trials across the fear conditioning and extinction studies, though the stimuli and US differed. Participants completed the STAI after the experiment.^†^^1^ During the acquisition phase, participants were presented with 24 trials, consisting of 12 CS + trials with 100% reinforcement (CS + paired with the US) and 12 CS- trials (presented without the US). The first two trials were a CS + followed by a CS- trial (or CS- followed by CS + ). Subsequent trials were presented in random order across participants with no more than two trials of either CS presented consecutively. A fixation cross was presented on the screen from CS offset to next CS onset. The intertrial interval (ITI) between each trial varied from between 16 to 30s. During the extinction phase, participants were presented with 24 trials consisting of 12 CS + (without the US) and 12 CS- trials in random order with no more than two trials of either CS presented consecutively.

#### Phase 2: EMA

Participants completed phase 2 within 4 weeks after completing phase 1. Data were collected via brief surveys embedded within text messages to participants' mobile phone. Study participants were assessed at random time-points, within pre-specified time-blocks, six times daily for seven days. Text messages were sent each morning (8:30–9:30), lunch (11:30–12:30), afternoon (2:30–3:30), evening (5:30–6:30), night-time (8:30–9:30pm), and bedtime (9:30–10:30pm). Surveys were closed within an hour of being sent.

### Response definitions and statistical analyses

#### Between-person measures

The primary between-person constructs of interest were the SCR indices. The magnitude of SCRs elicited during the presentation of each CS was scored within two latency windows; first interval responses (FIR) commencing within 1–5s after CS onset, reflecting the initial orienting response to the CS, (Öhman, 1983; Öhman & Bohlin, 1973; Prokasy, 1977), and late interval responses (LIR) occurring within 6–10s, providing a means of comparing responses to the US on CS + trials and US absence on CS- trials (Prokasy, 1977; Prokasy & Kumpfer, 1973). Separate average FIRs and LIRs were computed across trials for CS + and CS- during conditioning and extinction phases. See online Supplementary Section.

#### Between-person covariates

Analyses also controlled for STAI-trait. Given large variance in the STAI-T relative to other constructs, we completed a simple transformation (divided by two) to enhance model convergence. We further controlled for other relevant constructs at the person-level, including participant gender (0 = Female, 1 = Male), study stimuli (dogs = 0; shapes = 1), and EMA start day (Monday = 1 to Sunday = 7).

#### Within-person measures

The key within-person measures of interest were the EMA responses.

#### Momentary emotion

We tested for change in emotion by predicting anxious emotion at the current sampling moment (*T* = 0) while controlling for anxious emotion at the previous time point (*T* − 1). Thus, analyses characterise *changes* in anxiety, controlling for other momentary covariates.

#### Within-person covariates

*Momentary uplifts* and *Time of day,* a variable designating time of day for each momentary report (1 = morning to 6 = bedtime), were included in all models to control for potential confounds.

### Analytical plan

We ran a series of hierarchical linear models (HLM) to account for repeated measures of stress and emotion nested within-person (e.g. Hoffman and Rovine, 2007). HLM accounts for non-independence in the variables measured at each sampling moment and allows for separate estimation of within-person and between-person variance in outcomes. Within-person constructs are modelled at level 1 and between-person constructs are modelled at level 2. Level 1 variables were group-mean centred (centred on each participant's average for the week) and level 2 variables were grand-mean centred (on the sample's average) (Enders & Tofighi, 2007). With a total of 2, 142 possible responses (51 participants × 42 responses), 76% (*N* = 1637 data points) were available for analyses. We applied FIML estimation and all available data were employed to estimate missing data points (Enders, 2022).

Separate models were estimated for FIRs and LIRs to CS + and CS- trials during conditioning and extinction. Details of model building procedures are provided in the online Supplementary Section, along with relevant model equations. Model fit indices, including AIC, BIC, as well as associated within-person and between-person residual variances, are reported within results tables. Building models methodically, we sequentially modelled null, level one, two-level, and cross-level interaction models, with this final model which included the cross-level interaction term (skin conductance moderating the regression of emotion on stress) of critical interest and bolded in study tables. To account for multiple analyses, we applied a Benjamini Hochberg procedure for adjusted significance tests to manage the False Discovery Rate within sets of analyses (FDR; Benjamini & Hochberg, 1995). The FDR was applied within each table, with traditional *p* values reported, and those meeting FDR-corrected significance levels marked with an asterisk. Further sensitivity tests were run for all models, in which Study was entered as a control, as opposed to Stimuli as reported here. The same pattern of results emerged, and there were no significant findings associated with different study participation (See online Supplementary Section).

## Results

### Acquisition

#### First interval SCRs to CS- (CSM – FIR)

Building the models from level 1 predictors (left side of Table 1, level one model) of stressor, anxiety at T-1, uplift, and within-day timing of survey, we then modelled the predictive variation of the effect of experiencing a stressor on subsequent anxiety change based on theorised person-level constructs^2^ (two-level model column). Here, stimulus, study start day, trait anxiety, gender and the relevant skin conductance construct were entered into level 2. Next, we estimated a random coefficient model with the between-person skin conductance predictor (CSM-FIR) and gender. That is, two cross-level regression coefficients were simultaneously modelled (Cross-level interaction column, bolded). The regression coefficient for CSM-FIR was statistically significant (*b* = −0.10, *p* = 0.02) indicating that CSM-FIR moderated the anxiety on stress regression. Further, the regression coefficient was also significant for gender (*b* = −0.46, *p* = 0.02), indicating larger anxiety reactivity to stressors for females. Plots for all significant gender interactions are provided in online Supplementary Fig. S2.

**Table 1.** First interval skin conductance responses to CSM and CSP during acquisition and anxiety models

	*b* (s.e.) 95% CI	*b* (s.e.) 95% CI	*b* (s.e.) 95% CI	*b* (s.e.) 95% CI	*b* (s.e.) 95% CI	*b* (s.e.) 95% CI
	Null model	Level 1 model	Two-level model CSM-FIR	Cross-level interactions CSM-FIR	Two-level model CSP-FIR	Cross-level interactions CSP-FIR
Intercept	1.86 (0.12), *p* < 0.001 (1.63–2.09)	1.85 (0.11), *p* < 0.001 (1.63–2.09)	1.83 (0.09), *p* < 0.001* (1.66–2.00)	1.83 (0.09), *p* < 0.001* (1.66–2.00)	1.85 (0.09), *p* < 0.001* (1.65–2.01)	1.83 (0.09), *p* < 0.001* (1.65–2.01)
Within						
Time		−0.02 (0.01), *p* = 0.17 (0.04–0.01)	−0.01 (0.01), *p* = 0.34 (−0.04 to 0.01)	−0.01 (0.01), *p* = 0.25 (−0.04 to 0.01)	−0.01 (0.01), *p* = 0.34 (−0.04 to 0.01)	−0.02 (0.01), *p* = 0.25 (−0.04 to 0.01)
Uplift		−0.14 (0.06), *p* = 0.02 (−0.26 to −0.02)	−0.14 (0.07), *p* = 0.04 (−0.27 to 0.01)	−0.13 (0.07), *p* = 0.03 (−0.26 to 0.01)	−0.14 (0.07), *p* = 0.04* (−0.27 to 0.01)	−0.13 (0.06), *p* = 0.03* (−0.25 to 0.01)
Stressor		0.55 (0.09), *p* < 0.001 (0.37–0.73)	0.53 (0.10), *p* < 0.001* (0.34–0.71)	0.53 (0.09), *p* < 0.001* (0.35–0.71)	0.53 (0.01), *p* < 0.001* (0.34–0.71)	0.53 (0.09), *p* < 0.001* (0.35–0.71)
Anx Lagged		0.04 (0.02), *p* = 0.12 (−0.01 to 0.09)	0.04 (0.03), *p* = 0.10 (−0.01 to 0.09)	0.05 (0.03), *p* = 0.07 (−0.01 to 0.10)	0.04(0.03), *p* = 0.10 (−0.01 to 0.09)	0.05 (0.03), *p* = 0.06 (−0.01 to 0.10)
Variance within	1.40 (0.18), *p* < 0.001 (1.05–1.75)	0.67 (0.08), *p* < 0.001 (0.52–0.82)	0.66 (0.08), *p* < 0.001* (0.50–0.81)	0.64 (0.08), *p* < 0.001* (0.48–0.79)	0.66 (0.08), *p* < 0.001* (0.50–0.81)	0.64 (0.08), *p* < 0.001* (0.48–0.79)
Between						
Stimuli			0.39 (0.35), *p* = 0.26 (−0.29 to 1.08)	0.39 (0.35), *p* = 0.27 (−0.30 to 1.08)	0.39 (0.36), *p* = 0.29 (−0.33 to 1.10)	0.37 (0.37), *p* = 0.31 (−0.35 to 1.09)
Start Day			−0.13 (0.10), *p* = 0.22 (−0.33 to 0.02)	−0.13 (0.10), *p* = 0.22 (−0.33 to 0.07)	−0.15 (0.12), *p* = 0.21 (−0.37 to 0.08)	−0.15 (0.12), *p* = 0.21 (−0.38 to 0.08)
STAI-T			0.08 (0.02), *p* < 0.001* (0.05–0.11)	0.08 (0.02), *p* < 0.001* (0.05–0.11)	0.08 (0.02), *p* < 0.001* (0.05–0.11)	0.08 (0.02), *p* < 0.001* (0.05–0.11)
Gender			−0.27 (0.25), *p* = 0.28 (−0.75 to 0.22)	−0.27 (0.25), *p* = 0.29 (−0.75 to 0.22)	−0.33 (0.28), *p* = 0.24 (−0.88 to 0.22)	−0.33 (0.28), *p* = 0.24 (−0.89 to 0.22)
CSM/P-FIR			0.09 (0.03), *p* = 0.01* (0.03–0.16)	0.09 (0.03), *p* = 0.01* (−0.03 to 0.16)	0.04 (0.02), *p* = 0.04* (0.01–0.09)	0.04 (0.02), *p* = 0.05 (0.01–0.09)
Slope Gender				−0.46 (0.20), *p* = 0.02* (−0.85 to 0.08)		−0.43 (0.19), *p* = 0.02* (−0.80 to 0.07)
Slope CSM/P-FIR				−0.10 (0.04), *p* = 0.02* (−0.18 to 0.01)		−0.06 (0.03), *p* = 0.04* (−0.11 to 0.01)
Slope variance				0.17 (0.12), *p* = 0.16 (−0.07 to 0.41)		0.18 (0.13), *p* = 0.17 (−0.08 to 0.43)
Variance between		0.67 (0.16), *p* < 0.001 (0.35–0.98)	0.33 (0.07), *p* < 0.001* (0.19–0.46)	0.38 (0.07), *p* < 0.001* (0.19–0.46)	0.34 (0.07), *p* < 0.001* (0.20–0.48)	0.34 (0.07), *p* < 0.001* (0.20–0.49)
AIC	6205.23	4342.64	4408.30	4390.90	4411.16	4395.53
BIC	6216.39	4380.70	4483.91	4488.12	4486.76	4492.74

*Note.* Stressor = 0 = no bad event since last messaged, 1 = bad event since last messaged; Uplift = 0 = no uplift since last messaged, 1 = uplifting event since last messaged; Study stimuli = dogs = 0, shapes = 1; Start day = Monday = 1 to Sunday = 7; STAI-T = Trait anxiety score ÷ 2. Gender = 0 = female, 1 = male. Slope Coefficients represent the cross-level interactions (e.g. level-2 moderator of the level-1 anxiety on stressor regression). Two-level and cross-level model coefficients which met FDR-corrected significance level marked with an asterisk.

**Fig. 1.**
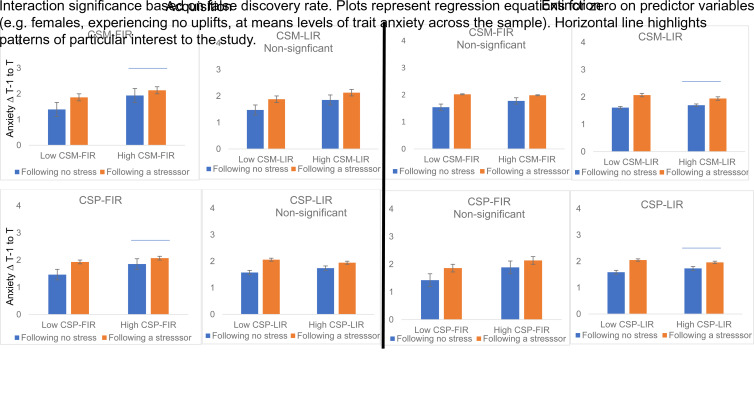
Anxiety change following no stress and a stressor as a function of low and high skin conductance responses during the early and late stage of the CS- (upper panels) and CS + lower panels during conditioning (left panels) and extinction (right panels).

We plotted and probed the simple slope of stress on anxiety change at one s.d. above and below the group mean for CSM-FIR to better characterise how the fear learning construct related to within-person changes in anxiety. The form of the interaction is described in Fig. 1 (upper left panel). Individuals lower in CSM-FIR (i.e. lower electrodermal reactivity to a safe cue during conditioning) correspondingly reported lower shifts in anxiety in safe moments during daily life (intercept = 1.63(0.10), *p* < 0.001) and showed significant increases in anxiety in response to daily stressors (*Z* = 3.67, *p* < 0.001). However, individuals high in electrodermal reactivity to a safe cue during conditioning reported relatively higher shifts in anxiety during safe moments in daily life (i.e. no stressor, intercept = 2.04(0.10), *p* < 0.001) and although they showed increased anxiety in the moments after a stressor, this was a non-significant change (*Z* = 1.16, *p* = 0.25); thus they remained elevated in anxiety across non-stressful and stressful moments in daily life.

#### Last interval SCRs to CS- (CSM – LIR)

We modelled the cross-level interaction terms (Table 2, set of columns on the left) to assess whether the predictive effect of stressor on anxiety change varied across individuals based on person-level predictors (CSM-LIR and gender). In this case, CSM-LIR was not a significant cross-level predictor of the impact of stress on anxiety change (*b* = −0.08, *p* = 0.24), though gender did predict significant variance in the level 1 effect. The pattern of the non-significant CSM-LIR interaction is characterised in Fig. 1.

**Table 2.** Last interval skin conductance responses to CSM and CSP during acquisition and anxiety models

	*b* (s.e.) 95% CI	*b* (s.e.) 95% CI	*b* (s.e.) 95% CI	*b* (s.e.) 95% CI	*b* (s.e.) 95% CI	*b* (s.e.) 95% CI
	Null model	Level 1 model	Two-level model CSM-LIR	Cross-level interactions CSM-LIR	Two-level model CSP-LIR	Cross-level interactions CSP-LIR
Intercept	1.86 (0.12), *p* < 0.001 (1.63–2.09)	1.85 (0.11), *p* < 0.001 (1.63–2.09)	1.83 (0.09), *p* < 0.001* (1.65–2.01)	1.83 (0.09), *p* < 0.001* (1.65–2.01)	1.83 (0.09), *p* < 0.001* (1.65–2.01)	1.83 (0.09), *p* < 0.001* (1.65–2.01)
Within						
Time		−0.02 (0.01), *p* = 0.17 (−0.04 to 0.01)	−0.01 (0.01), *p* = 0.34 (−0.35 to 0.01)	−0.01 (0.01), *p* = 0.26 (−0.04–0.01)	−0.01 (0.01), *p* = 0.34 (−0.04 to 0.01)	−0.01 (0.01), *p* = 0.26 (−0.04 to 0.01)
Uplift		−0.14 (0.06), *p* = 0.02 (−0.26 to 0.02)	−0.14 (0.07), *p* = 0.04 (−0.27 to 0.01)	−0.14 (0.06), *p* = 0.03 (−0.26 to −0.02)	−0.14 (0.07), *p* = 0.04* (−0.27 to −0.01)	−0.13 (0.06), *p* = 0.03* (−0.25 to −0.01)
Stressor		0.55 (0.09), *p* < 0.001 (0.37–0.73)	0.53 (0.10), *p* < 0.001* (0.34–0.71)	0.54 (0.10), *p* < 0.001* (0.35–0.73)	0.53 (0.10), *p* < 0.001* (0.34–0.71)	0.54 (0.09), *p* < 0.001* (0.36–0.72)
Anx Lagged		0.04 (0.02), *p* = .12 (−0.01 to 0.09)	0.04 (0.03), *p* = 0.10 (−0.01 to 0.09)	0.05 (0.03), *p* = 0.07 (−0.01 to 0.10)	0.04 (0.03), *p* = 0.10 (−0.01 to 0.09)	0.05 (0.03), *p* = 0.06 (−0.01 to 0.10)
Variance within	1.40 (0.18), *p* < 0.001 (1.05–1.75)	0.67 (0.08), *p* < 0.001 (0.52–0.82)	0.68 (0.08), *p* < 0.001* (0.50–0.81)	0.64 (0.08), *p* < 0.001* (0.48–0.79)	0.66 (0.08), *p* < 0.001* (0.50–0.81)	0.64 (0.08), *p* < 0.001* (0.48–0.79)
Between						
Shapes			−0.33 (0.36), *p* = 0.36 (−0.37 to 1.03)	0.32 (0.36), *p* = 0.38 (−0.39 to 0.1.02)	0.31 (0.35), *p* = 0.37 (−0.37 to 0.99)	0.28 (0.35), *p* = 0.42 (−0.41 to 0.97)
Start day			−0.11 (0.11), *p* = 0.30 (−0.33 to 0.10)	−0.12 (0.11), *p* = 0.29 (−0.33 to 0.10)	−0.16 (0.11), *p* = 0.16 (−0.38 to 0.06)	−0.16 (0.11), *p* = 0.16 (−0.38 to 0.06)
STAI-T			0.08 (0.02), *p* < 0.001* (0.05–0.12)	0.08 (0.02), *p* < 0.001* (0.05–0.12)	0.08 (0.02), *p* < 0.001* (0.04 to 0.11)	0.08 (0.02), *p* < 0.001* (0.04–0.11)
Gender			−0.26 (0.27), *p* = 0.33 (−0.78 to 0.27)	−0.27 (0.27), *p* = 0.32 (−0.80 to 0.26)	−0.41 (0.27), *p* = 0.13 (−0.94 to 0.13)	−0.41 (0.27), *p* = 0.13 (−0.95 to 0.12)
CSM/P-LIR			0.11 (0.06), *p* = 0.05 (0.01–0.22)	0.11 (0.06), *p* = 0.05 (0.01–0.22)	−0.01 (0.03), *p* = 0.74 (−0.05 to 0.07)	−0.01 (0.03), *p* = 0.76 (−0.05 to 0.07)
Slope gender				−0.41 (0.19), *p* = 0.03* (−0.78 to −0.03)		−0.48 (0.19), *p* = 0.02* (−0.82 to −0.08)
Slope CSM/P-LIR				**−0.08 (0.07), *p* = 0.24 (−0.23 to 0.06)**		**−0.06 (0.03), *p* = 0.03 (−0.12 to −0.01)**
Slope variance				0.20 (0.14), *p* = 0.14 (−0.07 to 0.47)		0.17 (0.12), *p* = 0.17 (−0.07 to 0.41)
Variance between		0.67 (0.16), *p* < 0.001 (0.35–0.98)	0.34 (0.07), *p* < 0.001* (0.21–0.47)	0.34 (0.07), *p* < 0.001* (0.21–0.47)	0.37 (0.08), *p* < 0.001* (0.22–0.52)	0.37 (0.08), *p* < 0.001* (0.22–0.52)
AIC	6205.23	4342.64	4410.76	4397.03	4413.61	4396.77
BIC	6216.39	4380.70	4486.37	4494.24	4489.22	4493.98

*Note.* Stressor = 0 = no bad event since last messaged, 1 = bad event since last messaged; Uplift = 0 = no uplift since last messaged, 1 = uplifting event since last messaged; Study stimuli = dogs = 0, shapes = 1; Start day = Monday = 1 to Sunday = 7; STAI-T = Trait anxiety score ÷ 2. Gender = 0 = female, 1 = male. Slope Coefficients represent the cross-level interactions (e.g. level-2 moderator of the level-1 anxiety on stressor regression). Two-level and cross-level model coefficients which met FDR-corrected significance level marked with an asterisk.

#### First interval SCRs to CS + (CSP – FIR)

Estimating the random coefficients model with level 2 predictors (CSP-FIR, gender) of the random slope indicated both the CSP-FIR and gender cross-level interaction regression coefficient were significant (Table 1, far right columns). The form of the CSP-FIR interaction (*b* = −0.06, *p* = 0.04) is described in Fig. 1, bottom left panel. Individuals at low levels of electrodermal reactivity to the CS + correspondingly reported lower anxiety across safe moments during daily life and showed significant increases in anxiety in response to daily stressors. In contrast, individuals who acquired more electrodermal reactivity at the early stage of CS + trials reported less change in anxiety from moments without relative to with a stressor, which was attributable to elevated anxiety to the moments without a stressor which were similar (i.e. non-significant) in response to stressors. Specifically, at low CSP-FIR scores (1 s.d. below the group mean), the intercept (intercept = 1.70(0.09), *p* < 0.001) and slope were significant (*Z* = 2.67, *p* = 0.008). At high CSP-FIR scores (1 s.d. above the group mean), the intercept was relatively higher (intercept = 1.96(0.10), *p* < 0.001) and the slope was non-significant (*Z* = 0.91, *p* = 0.36). Thus, greater electrodermal reactivity during early stages of CS + trials during conditioning predicted greater anxiety in response to safe moments in daily life that did not discriminate in relation to stressful events.

#### Last interval SCRs to CS + (CSP – LIR)

We next predicted variation in the effect of stressor on anxiety change via individual-level constructs of CSP-LIR and gender. As shown in Table 2 (right-most column), the cross-level interaction for CSP-LIR was *b* = −0.06(0.03), *p* = 0.03, but with the FDR applied, the interaction term no longer reached statistical significance. The pattern of the non-significant interaction for CSP-LIR is characterised within Fig. 1.

### Extinction

#### First interval SCRs to CS- (CSM – FIR)

We estimated the random coefficients model with CSM-FIR during extinction and gender as level 2 predictors of the random slope. As described in Table 3 (left), only the cross-level interaction for gender was statistically significant. The non-significant interaction pattern for CSM-FIR (*b* = −0.13, *p* = 0.06) is depicted in Fig. 1.

**Table 3.** First interval skin conductance responses to CSM and CSP during extinction and anxiety models

	*b* (s.e.) 95% CI	*b* (s.e.) 95% CI	*b* (s.e.) 95% CI	*b* (s.e.) 95% CI	*b* (s.e.) 95% CI	*b* (s.e.) 95% CI
	Null model	Level 1 model	Two-level model CSM-FIR	Cross-level interactions CSM-FIR	Two-level model CSP-FIR	Cross-level interactions CSP-FIR
Intercept	1.86 (0.12), *p* < 0.001 (1.63–2.09)	1.85 (0.11), *p* < 0.001 (1.63–2.09)	1.83 (0.09), *p* < 0.001* (1.65–2.01)	1.83 (0.09), *p* < 0.001* (1.65–2.01)	1.83 (0.09), *p* < 0.001* (1.65–2.00)	1.83 (0.09), *p* < 0.001* (1.65–2.00)
Within						
Time		−0.02 (0.01), *p* = 0.17 (−0.04 to 0.01)	−0.01 (0.01), *p* = 0.34 (−0.04 to 0.01)	−0.01 (0.01), *p* = 0.27 (−0.04 to 0.01)	−0.01 (0.01), *p* = 0.34 (−0.04 to 0.01)	−0.01 (0.01), *p* = 0.26 (−0.04–0.01)
Uplift		−0.14 (0.06), *p* = 0.02 (−0.26 to −0.02)	−0.14 (0.07), *p* = 0.04 (−0.27 to −0.01)	−0.14 (0.06), *p* = 0.03 (−0.26 to −0.01)	−0.14 (0.07), *p* = 0.04* (−0.27 to −0.01)	−0.14 (0.06) *p* = 0.03* (−0.26 to −0.01)
Stressor		0.55 (0.09), *p* < 0.001 (0.37–0.73)	0.53 (0.10), *p* < 0.001* (0.34–0.71)	0.53 (0.09), *p* < 0.001* (0.36–0.71)	0.53 (0.10), *p* < 0.001* (0.34–0.71)	0.54 (0.09), *p* < 0.001* (0.35–0.72)
Anx Lagged		0.04 (0.02), *p* = 0.12 (−0.01 to 0.09)	0.04 (0.03), *p* = 0.10 (−0.01 to 0.09)	0.05 (0.03), *p* = 0.07 (−0.01 to 0.10)	0.04 (0.03), *p* = 0.10 (−0.01 to 0.09)	0.05 (0.03), *p* = 0.07 (−0.01 to 0.10)
Variance within	1.40 (0.18), *p* < 0.001 (1.05–1.75)	0.67 (0.08), *p* < 0.001 (0.52–0.82)	0.66 (0.08), *p* < 0.001* (0.50–0.81)	0.64 (0.08), *p* < 0.001* (0.48–0.79)	0.66 (0.08), *p* < 0.001* (0.50–0.81)	0.64 (0.08), *p* < 0.001* (0.48–0.79)
Between						
Shapes			0.36 (0.35), *p* = 0.30 (−0.32 to 1.04)	0.32 (0.35), *p* = 0.37 (−0.37 to 1.01)	0.43 (0.35), *p* = 0.22 (−0.26 to 1.12)	0.42 (0.35), *p* = 0.23 (−0.27 to 1.11)
Start day			−0.15 (0.11), *p* = 0.19 (−0.37–0.07)	−0.15 (0.11), *p* = 0.18 (−0.38 to 0.07)	−0.14 (0.11), *p* = 0.22 (−0.36 to 0.08)	−0.14 (0.11), *p* = 0.21 (−0.36 to 0.08)
STAI-T			0.08 (0.02), *p* < 0.001* (0.04–0.11)	0.08 (0.02), *p* < 0.001* (0.04–0.11)	0.08 (0.02), *p* < 0.001* (0.05–0.11)	0.08 (0.02), *p* < 0.001* (0.05–0.12)
Gender			−0.42 (0.27), *p* = 0.12 (−0.95 to 0.11)	−0.43 (0.27), *p* = 0.12 (−0.95 to 0.11)	−0.36 (0.26), *p* = 0.17 (−0.87 to 0.16)	−0.36 (0.26), *p* = 0.17 (−0.88 to 0.16)
CSM/P-FIR			−0.05 (0.07), *p* = 0.46 (−0.08 to 0.18)	0.04 (0.07), *p* = 0.51 (−0.09 to 0.18)	0.10 (0.04), *p* = 0.01* (0.03–0.18)	0.10 (0.04), *p* = 0.01* (0.03–0.18)
Slope gender				−0.35 (17), *p* = 0.04* (−0.68 to −0.02)		−0.38 (0.18), *p* = 0.03 (−0.73 to −0.04)
Slope CSM/P-FIR				**−0.13 (0.07), *p* = 0.06 (−0.27 to 0.01)**		**−0.06 (0.04), *p* = 0.08 (−0.14 to 0.01)**
Slope variance				0.19 (0.13), *p* = 0.14 (−0.06 to –0.44)		0.20 (0.14), *p* = 0.15 (−0.07–0.48)
Variance between		0.67 (0.16), *p* < 0.001 (0.35–0.98)	0.36 (0.08), *p* < 0.001* (0.21–0.51)	0.36 (0.08), *p* < 0.001* (0.21–0.51)	0.33 (0.07), *p* < 0.001* (0.50–0.81)	0.33 (0.07), *p* < 0.001* (0.19–0.46)
AIC	6205.23	4342.64	4413.23	4397.45	4409.11	4395.37
BIC	6216.39	4380.70	4488.84	4494.66	4484.72	4492.58

*Note.* Stressor = 0 = no bad event since last messaged, 1 = bad event since last messaged; Uplift = 0 = no uplift since last messaged, 1 = uplifting event since last messaged; Study stimuli = dogs = 0, shapes = 1; Start day = Monday = 1 to Sunday = 7; STAI-T = Trait anxiety score ÷ 2. Gender = 0 = female, 1 = male. Slope Coefficients represent the cross-level interactions (e.g. level-2 moderator of the level-1 anxiety on stressor regression). Two-level and cross-level model coefficients which met FDR-corrected significance level marked with an asterisk.

#### Last interval SCRs to CS- (CSM – LIR)

Described in Table 4 (left), estimating the random coefficients model with CSM-LIR during extinction and gender as the level 2 predictors, only the CSM-LIR cross-level interaction was statistically significant (*b* = −0.10, *p* = 0.03). The top right panel of Fig. 1 describes the interaction**.** As shown, individuals at low CSM-LIR levels during extinction showed significant increases in anxious reactivity in response to stressors. Whereas individuals higher on electrodermal reactivity to the CS- during extinction were generally the same across both non-stressful and stressful moments. Probing of the simple slopes indicate that for individuals at low levels of CSM-LIR during extinction (1 s.d. group mean), the intercept was slightly lower (intercept = 1.83(0.09), *p* < 0.001) and the slope was steeper (*Z* = 4.88, *p* < 0.001) whereas for individuals scoring high (1 s.d. above the group mean) in CSM-LIR during extinction, the intercept was significant and slightly higher (intercept = 1.83(0.08), *p* < 0.001) and the slope was not significant (*Z* = 1.73, *p* = 0.084). Thus, electrodermal reactivity during the later stages of responding to safe stimuli during extinction predicted greater anxiety across safe moments in daily life that did not discriminate in relation to stressful events.

**Table 4.** Last interval skin conductance responses to CSM and CSP during extinction and anxiety models

	*b* (s.e.) 95% CI	*b* (s.e.) 95% CI	*b* (s.e.) 95% CI	*b* (s.e.) 95% CI	*b* (s.e.) 95% CI	*b* (s.e.) 95% CI
	Null model	Level 1 model	Two-level model CSM-LIR	Cross-level interactions CSM-LIR	Two-level model CSP-LIR	Cross-level interactions CSP-LIR
Intercept	1.86 (0.12), *p* < 0.001 (1.63–2.09)	1.85 (0.11), *p* < 0.001 (1.63–2.09)	1.83 (0.09), *p* < 0.001* (1.65–2.01)	1.83 (0.09), *p* < 0.001* (1.65–2.01)	1.83 (0.09), *p* < 0.001* (1.65–2.01)	1.83 (0.09), *p* < 0.001* (1.65–2.01)
Within						
Time		−0.02 (0.01), *p* = 0.17 (−0.04 to 0.01)	−0.01 (0.01), *p* = 0.34 (−0.04 to 0.01)	−0.01 (0.01), *p* = 0.27 (−0.04 to 0.01)	−0.01 (0.01), *p* = 0.34 (−0.04 to 0.01)	−0.01 (0.01), *p* = 0.27 (−0.04 to 0.01)
Uplift		−0.14 (0.06), *p* = 0.02 (−0.26 to −0.02)	−0.14 (0.07), *p* = 0.04 (−0.27 to 0.01)	−0.14 (0.06), *p* = 0.03* (−0.26 to −0.02)	−0.14 (0.07), *p* = 0.04* (−0.27 to −0.01)	−0.14 (0.06), *p* = 0.02* (−0.27 to −0.02)
Stressor		0.55 (0.09), *p* < 0.001 (0.37–0.73)	0.53 (0.10), *p* < 0.001* (0.34–0.71)	0.55 (0.09), *p* < 0.001* (0.36–0.73)	0.53 (0.10), *p* < 0.001* (0.34–0.71)	0.54 (0.09), *p* < 0.001* (0.36–0.72)
Anx Lagged		0.04 (0.02), *p* = 0.12 (−0.01 to 0.09)	0.04 (0.03), *p* = 0.10 (−0.01 to 0.09)	0.05 (0.03), *p* = 0.07 (−0.01 to 0.10)	0.04 (0.03), *p* = 0.10 (-0.01–0.09)	0.05 (0.03), *p* = 0.07 (−0.01 to 0.10)
Variance within	1.40 (0.18), *p* < 0.001 (1.05–1.75)	0.67 (0.08), *p* < 0.001 (0.52–0.82)	0.66 (0.08), *p* < 0.001* (0.50–0.81)	0.64 (0.08), *p* < 0.001* (0.48–0.79)	0.66 (0.08), *p* < 0.001* (0.50–0.81)	0.64 (0.08), *p* < 0.001* (0.48–0.79)
Between						
Shapes			0.29 (0.36), p = 0.41 (−0.40 to 0.97)	0.24 (0.36), *p* = 0.50 (−0.47 to 0.96)	0.30 (0.34), *p* = 0.38 (−0.37 to 0.96)	0.27 (0.35), *p* = 0.44 (−0.41 to 0.95)
Start day			−0.16 (0.12), *p* = 0.17 (−0.38 to 0.07)	−0.16 (0.12), *p* = 0.16 (−0.39 to 0.06)	−0.15 (0.12), *p* = 0.18 (−0.38 to 0.07)	−0.16 (0.12), *p* = 0.17 (−0.39 to 0.07)
STAI-T			0.08 (0.02), *p* < 0.001* (0.04–0.11)	0.08 (0.02), *p* < 0.001* (0.04–0.11)	0.08 (0.02), *p* < 0.001* (0.04–0.11)	0.08 (0.02), *p* < 0.001* (0.04–0.11)
Gender			−0.42 (0.27), *p* = 0.12 (−0.95 to 0.11)	−0.43 (0.27), *p* = 0.12 (−0.96 to 0.11)	−0.41 (0.27), *p* = 0.13 (−0.95 to 0.12)	−0.42 (0.27), *p* = 0.12 (−0.96 to 0.11)
CSM/P-LIR			−0.01 (0.06), *p* = 0.96 (−0.11 to 0.12)	−0.01 (0.06), *p* = 0.99 (−0.11 to 0.11)	−0.02 (0.07), *p* = 0.78 (−0.12 to 0.16)	0.02 (0.07), *p* = 0.80 (−0.12 to 0.16)
Slope gender				−0.36 (0.17), *p* = 0.04 (−0.69 to −0.02)		−0.36 (0.17), *p* = 0.04* (−0.70 to −0.02)
Slope CSM/P-LIR				−0.10 (0.05), *p* = 0.03* (−0.20 to −0.01)		−0.16 (0.07), *p* = 0.02* (−0.30 to −0.03)
Slope variance				0.19 (0.13), *p* = 0.15 (−0.07–0.45)		0.18 (0.13), *p* = 0.17 (−0.08 to 0.44)
Variance between		0.67 (0.16), *p* < 0.001 (0.35–0.98)	0.37 (0.08), *p* < 0.001* (0.22–0.52)	0.37 (0.08), *p* < 0.001* (0.22–0.52)	0.37 (0.08), *p* < 0.001* (0.22–0.51)	0.37 (0.08), *p* < 0.001* (0.22–0.52)
AIC	6205.23	4342.64	4413.71	4398.40	4413.64	4397.45
BIC	6216.39	4380.70	4489.32	4495.61	4489.25	4494.66

*Note.* Stressor = 0 = no bad event since last messaged, 1 = bad event since last messaged; Uplift = 0 = no uplift since last messaged, 1 = uplifting event since last messaged; Study stimuli = dogs = 0, shapes = 1; Start day = Monday = 1 to Sunday = 7; STAI-T = Trait anxiety score ÷ 2. Gender = 0 = female, 1 = male. Slope Coefficients represent the cross-level interactions (e.g. level-2 moderator of the level-1 anxiety on stressor regression). Two-level and cross-level model coefficients which met FDR-corrected significance level marked with an asterisk.

#### First interval SCRs to CS + (CSP – FIR)

Characterised within Table 3 (right), the regression coefficients for the cross-level interactions (CSP-FIR and gender) were non-significant. The pattern associated with the non-significant CSP-FIR interaction (*b* = −0.06, *p* = 0.08) is characterised in Fig. 1.

#### Last interval SCRs to CS + (CSP – LIR)

The CSP-LIR coefficient for the cross-level interaction was statistically significant (Table 4, right), as was the coefficient for gender. Examining the negative CSP-LIR coefficient (*b* = −0.16, *p* = 0.02) suggests that for participants with higher CSP-LIR during extinction, the impact of stressor on subsequent anxiety change was smaller than expected based on only the direct effect. Figure 1 (bottom right panel) describes the interaction. Individuals high on CSP-LIR during extinction were generally similar following moments when no stressor occurred as when moments followed a stressor. However, individuals low on CSP-LIR during extinction increased their anxious responses in relation to stressors. Hence individuals high on CSP-LIR remained similarly elevated in anxiety across non-stressful and stressful moments in daily life. Probing of the simple slopes showed that for individuals at low levels of CSP-LIR during extinction, the intercept was relatively lower (intercept = 1.83(0.09), *p* < 0.001) and the slope was significant (*Z* = 4.87, *p* < 0.001), whereas for individuals scoring high on CSP-LIR, the intercept was slightly higher and the slope was not significant (intercept = 1.83(0.08), *p* < 0.001; slope *Z* = 1.73, *p* = 0.08). Thus, high electrodermal reactivity during late stages of the CS + during extinction (i.e. absence of the US) predicted relatively similar reactivity across non-stressful moments as stressful moments in daily life.

## Discussion

This is the first empirical study, to our knowledge, to examine whether fear learning processes translate to real world, ‘in the moment’ experience of anxiety in daily life. Findings indicated that larger first intervals SCRs to threat conditioned stimuli (CS + ) and safe stimuli (CS-) during acquisition predicted sustained elevations in anxiety across non-stressful moments and stressful moments in daily life. Late interval SCRs during acquisition did not condition the relation between experiencing stressors and momentary anxiety changes in daily life. Likewise, during extinction, first interval SCRs did not condition the relation between stressors and anxiety changes. However, larger last interval SCRs to safe stimuli (CS-) and threat stimuli (CS + ) (i.e. absence of the US) were predictive of sustained elevations in anxiety across non-stressful moments and stressful moments in daily life. Study findings were above and beyond the explanatory role of trait anxiety.

Fear learning models and empirical evidence suggest that individuals who more readily acquire heightened conditioned fear responses extrapolate these associations to safe stimuli and have trouble tamping down conditioned responses with new information indicating safety (Scheveneels et al., 2021; Vervliet, Craske, & Hermans, 2013; Waters et al., 2014). The key contribution of this work is that because individuals showing heightened fear responses generalise such heightened associative processes to both daily stressful *and* non-stressful events (the latter of which do not require an emotionally reactive response), they are at increased risk for anxiety disorders and treatment resistance (Guthrie & Bryant, 2006; Lommen et al., 2014; Orr et al., 2012). Indeed, we found that electrodermal reactivity to a threat conditioned stimulus (i.e. the CS + ), overgeneralisation to safe stimuli (i.e. CS-) and persistent electrodermal elevations during extinction (when both the CS- and CS + are now safe) predicted real-world overgeneralisation of anxious reactivity to benign (e.g. safe) moments in which no stressful events occurred in daily life. This suggests that vulnerability to acquiring and generalising conditioned fear responses, and difficulty inhibiting these responses with new safety information, may be reflective of aberrations in underlying mechanisms by which a wide range of benign situations in daily life become associated with threat and anxiety (Waters & Craske, 2016).

One potential mechanism for this effect is that enhanced acquisition and generalisation of conditioned physiological reactivity is associated with the propensity to evaluate other events in daily life as similarly threatening as genuine stressors. This would result in minimal discrimination in the evaluation of safe and threat situations and thus similar shifts in anxiety in response to safe situations and genuine threats in daily life (Waters & Craske, 2016). The influence of fear learning experiences on elaborative processing can also be interpreted as consistent with the model of emotional contrast avoidance in anxiety (e.g. Newman & Liera, 2011; Newman et al., 2019). That is, elevated reactivity to threat and safe stimuli may reflect a baseline hypersensitivity to upward shifts in emotion to negative events that persists across non-stressful situations. By sustaining negative emotion, worry enables the avoidance of negative emotional shifts in response to unexpected stressors. This may explain the observation that elevated last interval SCRs to the CS- in extinction (i.e. the interval temporally associated with when the US is delivered on CS + trials during acquisition) predicted less change in anxiety in response to genuine stressors in daily life. That is, maintaining reactivity to a safe stimulus that has a very weak association with threat (i.e. same timing of the US) as well as to a threat stimulus (i.e. the CS + ) that is now safe is related to daily life patterns wherein less of an upward shift in anxiety is experienced in response to genuine stressors. Thus, the unpleasant experience of moving from neutral to negative emotion can be avoided by sustained, elevated worry (Newman & Llera, 2011). Further studies are required that include momentary sampling of stressors and situational evaluations alongside emotions across daily life. This would permit the examination of the extent to which aberrant fear learning and extinction influences elevated emotional reactivity in daily life via the impact on threat evaluation and worry (Newman & Llera, 2011; Waters & Craske, 2016).

Although in need of replication, the present findings may have important experimental and clinical implications. First, they highlight that experimental analogue tasks have the potential to elucidate aberrant processes under baseline conditions that are distinct from pre-anxiety baseline conditions (Newman et al., 2019). They also suggest that mere exposure to fear stimuli alone during exposure therapy is insufficient to attenuate generalised reactivity in daily life. If the propensity for heightened conditioned fear and generalisation is underpinned by biases in elaborative processes such as evaluations and worry, then exposure therapy may be enhanced by the inclusion of exposure to threat outcomes, not just the stimulus. This may enhance the ability to tolerate shifts in distress rather than avoid the stimulus and sustain distress through worrying (Newman et al., 2019). It may also be beneficial to couple exposure trials to the stimulus and outcome with strategies such as memory rehearsal to maximise the retention of new learning, such as memory rehearsal to maximise safe-threat stimulus discrimination and prevent overgeneralisation (Craske et al., 2009; Waters & Craske, 2016).

Study limitations also merit consideration. First, we did not collect data on ethnicity, and this would be an important consideration for future work. Second, our sample size is relatively small. Additional research would benefit from both a larger sample size and inclusion of clinical samples. Third, we included fear conditioning and extinction phases only. Future studies should include an extinction retest phase or return of fear manipulation (e.g. renewal) to examine longer-term and relapse mechanisms as predictors of daily emotion reactivity. Finally, we explored anxiety, stressors, and uplifts across the course of a week. Exploration of the impact on other emotions such as depressed mood and anger would be important next steps.

Our findings show that aberrant electrodermal (skin conductance) reactivity during laboratory-based Pavlovian fear conditioning and extinction tasks is associated with increased anxious reactivity during benign moments of everyday life and this same level of reactivity translates to stressful moments. These findings highlight the validity of laboratory-based measures of fear learning impairments in predicting anxious reactivity in daily life and warrants further study, in particular, the inclusion of evaluations of everyday events and stressors.

## Footnotes

† The notes appear after the main text.

## Notes

1 They also provided subjective ratings of CS valence and arousal and anxiety before and after each phase and SCRs were measured during each trial.

2 All models were built up methodically. Random slopes were also tested for uplifts, which resulted in increased BIC values and little change in unexplained level 1 variance. Cross-level interaction terms were introduced independently for each covariate to ensure models were correctly specified. Neither STAI, Study Stimuli, nor Start Day were significant level 2 predictors of the within-person effects.

## Supplementary material

For supplementary material accompanying this paper visit https://doi.org/10.1017/S0033291722002379. click here to view supplementary material
