# Supplementary material for: Fear learning and extinction predicts anxiety in daily life: a study of Pavlovian conditioning and ecological momentary assessment
Source: Psychol Med. 2022 Sep 12;53(11):5301–11. doi: 10.1017/S0033291722002379 (PMC10476067; doi:10.1017/S0033291722002379)
Supplement: Supplementary file 1 [file S0033291722002379sup001.docx]

**Supplementary Section**

Fear learning and extinction predicts anxiety in daily life: A study of Pavlovian conditioning and ecological momentary assessments

**Full Methods**

**Measures**

***Phase one: Conditioning and extinction task***

**Stimulus materials.** There were two separate stimuli uses across four studies which contributed to this larger project. Two studies (#1 and #2) used geometric shapes paired with a scraping sound. Here, the geometric shapes were a pastel cream triangle and pastel pink trapezoid as the CSs. Shapes were counterbalanced across participants and presented for 8 s on a black screen. Geometric shapes were presented on a Dell 15 ̋ colour laptop at approximately 1 m distance. The US was a 3s recording of a three-pronged garden fork scraped over slate (see Neumann & Waters, 2006; Waters et al., 2017) that was presented through Sony headphones at a peak 100 dB. The US was presented only during conditioning with the CS+ 100% of the time for 1s at 7s following CS+ onset. Two studies (#3 and #4), used photographs of dogs paired with a dog growl and scream. The dog photographs served as the CSs. The two dogs were counterbalanced across participants and appeared on the screen for 11s. The US was a 3s aversive sound of a dog growling and a woman screaming reaching 100 dB. The growl commenced 0.5s before the scream and then both sounds were presented simultaneously for the remaining 2.5s. The US was presented at 8s after CS onset on 100% of CS+ trials. Information is provided in Supplementary Table 1 related to the descriptives associated with each stimulus. As described, there were no differences in gender, age, or STAI trait across the stimuli. Nor were there differences in precent correct. Although the dogs stimuli was rated as more intense and less unpleasant than the shapes stimuli, they produce the same effect. That is, neither stimuli nor study covariates emerge as significant within any of our models (stimuli non-significant within main study findings; study non-significant with supplementary findings).

In terms of the four studies, all were fear learning and extinction task studies. Within the fear conditioning lab, two different stimuli are used, which follow the exact same protocol, and vary only in the stimuli-pairing. Participants took part in a specific study based simply on the timing of their lab participation, with studies running consecutively. With the onset of funding support, we began pairing the ESM protocol with the studies running in the lab—all of which examined skin conductance in relation to fear learning, and all with similar trial numbers and length. The individual studies hence focused on specific questions within this existing paradigm. For the current project, we used the control condition for each of the four studies (it is the participants in the other conditions who received a different manipulation). That is, across conditions, participants all received exactly the same instructions, numbers of trials, and provided the same dependent variable measures, the only difference was for the stimuli.

Indeed, examination of potential study differences across fear learning constructs via a series of one-way ANOVA, findings generally showed no differences across studies in relation to Acquisition: CSM-FIR (*F*(3, 47) = .25, *p* = .86; CSP-FIR (*F*(3, 47) = 1.30, *p* = .29; CSM-LIR *F*(3, 47) = .85, *p* = .472; or Extinction: CSM-FIR *F*(3, 47) = 1.01, *p* = .40; CSP-FIR *F*(3, 47) = .99, *p* = .60; CSM-LIR *F*(3, 47) = .64, *p* = .60; CSP-LIR *F*(3, 47) = .41, p = .75. Although overall group differences emerged on one skin-conductance construct during acquisition, CSP-LIR *F*(3, 47) = 4.45, *p* = .008. Follow up Tukey HSD analyses indicated no significant differences in comparing individual studies, as follows: Study 1 and 2 Mean_Diff_ = .11(.19), *p* = .94; Study 1 and 3 Mean_Diff_ = -.31(.13), *p* = .08; Study 1 and 4 Mean_Diff_ = -.03(.17), *p* = .99; Study 2 and 3 Mean_Diff_ = -.41(.16), *p* = .06; Study 2 and 4 MeanDiff = -.14(.19), *p* = .89; or Study 3 and 4 Mean_Diff_ = .27(.16), *p* = .18.

***Skin Conductance Responses (SCR).*** SCR were recorded using two self-adhesive isotonic electrodes (Biopac systems EL507) attached to the thenar and hypothenar eminences of the non-dominant hand. They were acquired using a Biopac data acquisition system (Model MP150) with a sampling frequency of 2000Hz via an EDA100C amplifier. Data were analysed using AcqKnowledge software Version 5.0. Respiration was recorded using a Biopac TSD201 transducer connected to an RSP100C respiration amplifier, to monitor for respiratory influences on SCRs. Participants rated the CS+ and CS- before acquisition, after acquisition, after extinction, and after retest on the computer screen. After acquisition, they also rated the US post-acquisition for intensity and pleasantness and contingency awareness was assessed. Data not reported as these ratings have not been found to be associated with anxiety differences. Data were scored manually, with the trough and apex being defined as the low point and high point of the curve, respectively.

Correlations between the skin conductance responses and trait anxiety, and the mean, standard deviation, and range of the skin conductance constructs were as follows: **Acquisition**: CSM-FIR 2.45(2.31), 0 -8.81, *r*(44) = -.16, *p* = .27; CSP-FIR 3.39(3.28), 0 -11.90, *r*(44) = -.14, *p* = .33; CSM-LIR 1.69(1.48), 0 – 5.87, *r*(44) = -.267, *p* = .058; CSP-LIR 4.72(3.27), .52-12.46, *r*(44)= .07, *p* = .63. **Extinction**: CSM-FIR 1.86(1.58), 0 – 6.09, *r*(44) = -.266, *p* = .059; CSP-FIR 2.26(2.23), 0 -11.6, *r*(44) = -.313, *p* = .03; CSM-LIR 1.54(1.59), 0 -8.92, *r* (44) = -.274, *p* = .052; and CSP-LIR 1.29 (1.15); 0 - 4.25, r(44) = -.017, *p* = .91.

**Phase two**

***Phase two: Ecological Momentary Assessment (EMA)***

Up to eight EMA items were presented in total, based on our previous EMA work with adolescents and young adults (Modecki et al., 2022; Uink et al., 2017), with the goal of a very brief survey that would lend itself to high compliance. Four emotions were tapped: Happy, Sad, Anxious, and Angry, based on previous EMA studies (Schneiders et al., 2006; Silk et al., 2003). Participants were then asked if they had experienced a stressor or negative event since the last message Yes/No (Schneiders et al., 2006). If they responded Yes, the question branched to a rating of the stressor (1-5) (Schneiders et al., 2006). All participants were then asked if they had experienced a good event or uplift since the last message Yes/No. If they responded yes, again, the question then branched to a rating of the good event (1-5) (Uink et al., 2018). Overall, each survey took roughly 2-3 minutes to complete.

***Momentary emotion****.* Participants were asked *“Please indicate how _____ you feel right now?”* at each sampling moment. Participants rated how happy, sad, angry, and anxious they were feelings in line with previous studies (1 = Not at all, 5 = Very much) (Modecki et al., 2022; Schneiders et al., 2006; Silk et al. 2003). For the current study, we focused on anxious emotions in relation to stress responses. Momentary anxiety scores ranged from 1-5, *M* = 1.89, *SD* = 1.19. Momentary anxiety response rates ranged from 50 to 100% of the 42 possible responses with 82% of participants responding to 80% or more time points (87% average).

***Momentary stress****.* Participants were asked “Since the last set of questions, has anything negative or stressful happened to you?” at each sampling moment (Schneiders et al., 2006; Uink et al., 2017). The question format ensured that participants reported on recent stressors in the last several hours. Because we were seeking to reduce participants’ response burden within the EMA, we did not ask participants to describe the event via open-ended reports (Modecki et al., 2019; van Roekel et al., 2019). In previous research we have found these queries are often left blank or are interpretable (Uink et al., 2017). It is possible that participants interpreted this item heterogeneously, though trait anxiety did not differentiate number of stressors reported across the week within our sample (*r*(44), = .03, *p* = .84). Momentary analyses also indicated good validity, and the item was positive correlated with momentary anger (*r* (1920) = .41, *p* < .001) and negatively correlated with momentary happiness (*r* (1920) = -.20, *p* < .001). Participants reported stressful events at approximately 12% of all sampling moments. Responses were dummy coded (0 = no bad event since last messaged, 1 = bad event since last messaged). Momentary stressors response rates likewise ranged from 50 to 100% with 79% of participants responding to 80% or more time points (87% average response rate).

***Momentary uplifts.*** Participants also reported on momentary uplifts, in order to control for these experiences in all models. At each sampling moment, individuals were asked, “Since the last set of questions, has anything positive or very good happened to you?” (Schneiders et al., 2006; Uink et al., 2018). Participants reported positive events/uplifts at approximately 18% of all sampling moments. Momentary uplifts response rated ranged from 50 to 100%, and 79% of participants responded to 80% or more of the 42 time points (87% average response rate).

***Time of day***. We coded participant responses based on time of their survey. From 1 = morning (8:30-9:30) to 7 = bed-time (9:30-10:30pm). This was used simply as a control variable, similar to many EMA studies, given that emotions fluctuate within and across days (Smyth et al., 2009).

**Procedure**

***Phase 2: EMA.*** Participants completed phase 2 within 4 weeks after completing phase 1. Data were collected via brief surveys embedded within text messages to participants’ mobile phone. Each text message was designed so that participants could respond directly within each text, rather than a link to a separate survey site. Each text survey was closed within an hour of the text being sent. Participants received payment via a store gift card for completing the EMA phase with the final value based on $1 for every message they replied to with the maximum value being a $42 gift card.

**Response definitions**

**Between Person Measures.** The primary between-person constructs of interest were the SCR indices. The magnitude of SCRs elicited during the presentation of each CS was scored within two latency windows; first interval responses (FIR) commencing within 1-5s after CS onset, reflecting the initial orienting response to the CS, (see Öhman, 1983; Öhman & Bohlin, 1973; Prokasy, 1977), and late interval responses (LIR) occurring within 6-10s, providing a means of comparing responses to the US on CS+ trials and US absence on CS- trials (Prokasy, 1977; Prokasy & Kumpfer, 1973). Responses were scored trial-by-trial if they began in these onset windows and comprised the difference between the trough and apex of the curve expressed in microsiemens (mS). All trials with artefacts due to respiration or movement were removed (less than 10% of all trials). Separate average FIRs and LIRs were computed across trials for CS+ and CS- during conditioning and extinction phases. The relevant SCR construct was entered into level two for each set of analyses.

**Analytic Plan**

With a total of 2, 142 possible responses (51 participants x 42 responses), 76% (N = 1,637 data points) were available for analyses. Missing data on momentary anxiety was unrelated to any other constructs in the model (e.g. any of the 8 skin conductance constructs, gender, start day), with correlations ranging from .01 (*p* = .94) to .12 (*p* = .39). Missing data on momentary stressors was likewise unrelated any other constructs in the model, with correlations ranging from .01 (*p* = .94) to .13 (*p* = .36). Finally missing data on momentary uplifts was unrelated to any other model constructs, with correlation ranging from .0001 (*p* = .99) to .13 (*p* = .36) Further, we applied FIML estimation and all available data were employed to estimate missing data points, in line with best practice (Enders, 2010). There were no missing data points for the between-level variables with the exception of STAI. Here. we followed recommendations by Muthén and Muthén (e.g. May 5, 2015) <http://www.statmodel.com/discussion/messages/12/162.html?1506635289> and brought the STAI covariate into the FIML model. We included Montecarlo integration for model execution.

Models were built up from an intercept-only model to a model including the relevant cross-level interaction terms. Following the intercept-only model, we estimated a model with fixed lower-level explanatory variables. We then added higher-level explanatory variables at the between person level. Next, we estimated a series of random coefficient models and tested for random slope variation for momentary stressor (as well as a check for random slope variation for momentary uplifts). After determining that stress pointed to likely variance between people, the variance component was added to the model. At the final step, we tested cross-level interactions between explanatory person-level variables and the random slope predicting anxiety change. Person-level covariates were tested independently before all significant interaction terms were added to the final model. Model fit indices, including AIC, BIC, deviance, and -2LL, as well as associated within person and between person residual variances, are reported within results tables.

To account for multiple analyses, we applied a Benjamini Hochberg procedure for adjusted significance tests to manage the False Discovery Rate within sets of analyses ([FDR; Benjamini and Hochberg, 1995](https://www.ncbi.nlm.nih.gov/pmc/articles/PMC6953732/#R5)). The FDR was applied within each table, with traditional *p* values reported, and those meeting FDR-corrected significance levels marked with an asterisk. Further Sensitivity Tests were run for all models, in which Study was entered as a control variable, as opposed to Stimuli as reported in the main manuscript. The same pattern of results for skin conductance measures emerged, and there were no significant findings associated with different study participation. Below, we describe in greater detail the findings reported in the manuscript, including describing, plotting, and probing cross-level interactions found for gender—that is, the gender by stressor predicting emotion regression equation. We also provide full tables associated with the Sensitivity Tests (Supplementary Tables 2-5).

In terms of model equations, first the level-1 model was estimated. Here, the participant’s emotion reactivity modelled as a function of the predicted average (intercept value) of emotion for the week (β0j), emotion at t-1 (β1j), whether they reported a stressor ($\beta2j$) or positive event ($\beta3j$) at that moment, time of day as a control ($\beta4j$), and variation around their momentary anxiety average ($e_{ij}$).

**Level 1 Model Equation**:

$${Anxiety Change}_{ij}= \beta0j+ \beta1j \left( anxiety t-1 \right)+ \beta2j \left( stressor \right)+ \beta3j\left( uplift \right)+ \beta4j \left( time of day \right)+ e_{ij}$$

We next modelled the Level 1 intercepts at Level 2 of the model. Here, individual’s average anxiety change was modelled as a function of the average level of anxiety across the sample ($\gamma00$), plus the relevant skin conductance construct ($\gamma01$), participant gender ($\gamma02$), trait anxiety (γ03), and controls of study start day ($\gamma04$) and stimuli ($\gamma05$).

**Level 2 Model Equation:**

$$\beta0j= \gamma00+\gamma01 \left( skin conductance \right)+ \gamma02 \left( gender \right)+ \gamma03\left( STAI-T \right)+ \gamma04 \left( start day \right)+ \gamma05\left( stimuli \right)+ \mu0j$$

Next, to test for between-person differences in anxiety change, we allowed the within-person slope of anxiety on stressor to vary across individuals (i.e., The random-slope component of the model). (Random slopes were also tested for uplifts, which resulted in increased BIC values and little change in unexplained level 1 variance). Following this, we regressed the within-person slope onto skin conductance and gender. Notably, cross-level interaction terms were introduced independently for each covariate to ensure models were correctly specified. Neither STAI, Start Day, nor Study Stimuli were significant level 2 predictors of the within-person effects. Here, γ10 represents the pooled slope coefficient of the within-person relation between anxiety and stressor and γ11 and $\gamma12$ represent cross-level interactions on the level-1 slope and μ1j represents a participant’s deviation around the average slope.

**Additional Cross-Level Interaction Equation:**

$$\beta1j= \gamma10+\gamma11 \left( skin conductance \right)+ \gamma12 \left( gender \right)+ \mu1j$$

**Full Results**

**Base model results**. Correlations between time points for anxiety within individuals was ICC = .48 indicating 48% of the variance in anxiety occurred at the person level. The design effect was 20.68, justifying our use of multilevel models (Table 1, first column on the left). Next, level 1 variables of stressor yes/no, uplift yes/no, lagged anxiety, and the time of day control variable were added to the model. As described in Table 1 of the manuscript (moving rightwards, second column from the left), at level 1, an uplift was associated with a .14 decrease in anxiety, and a stressor was associated with a .55 increase in anxiety. The intercept of the predicted model (the average momentary anxiety score across all participants) for anxiety was 1.85, 95%CI [1.63 ─ 2.09] and significantly different from zero, *p* < .001. After accounting for stressors, uplifts, prior emotion, and time of day, the estimated variation in intercepts across participants was .67, 96%CI [.52 ─ .82] and significant (p < .001).

**CSM - FIR.** We next examined a two-level fixed effects model for CSM-FIR (Table 1, third column from left), which indicated that for every two scale-points a participant scored higher on the STAI-T (recall that STAI-T was transformed), their average anxiety score reported across the week increased by .08 (*b* = .08(.02), *p* < .001). There was also a positive main effect for CSM-FIR (*b* = .09(.03), *p* = .01), which indicated that for every point increase in CSM-FIR, their average anxiety score across the week increased by .09.

We then modelled the random slope for the within-cluster effect of stressor on pre- to post- stress anxiety. The within-person effect of stressor on increased anxiety remained significant (*b* = .55(.10), *p* < .001) and this effect appeared to vary across individuals, given a marginal slope variance (t = 1.54; Asparouhov, 2017) and improved model fit as indicated by -2LL difference test, reduced AIC, BIC, and level 1 residual variance. Notably, it is not a requirement that significant slope variance be indicated in order to test for cross-level interactions, as improved model fit (for example, from interaction terms) coincides with enhanced power to test effects (Snijders & Bosker, 1999).

As a result, we next modelled the predictive variation of the effect of experiencing a stressor on subsequent anxiety change based on theorized person-level constructs. All models were built up methodically. Random slopes were also tested for uplifts, which resulted in increased BIC values and little change in unexplained level 1 variance. Cross-level interaction terms were introduced independently for each covariate to ensure models were correctly specified. Neither STAI, Start Day, nor Study Stimuli were significant level 2 predictors of the within-person effects.

We estimated a random coefficient model with the between-person predictor CSM-FIR, alongside the other level 2 predictor which showed predictive effects (gender). Thus, two cross-level regression coefficients were simultaneously modelled. The regression coefficient for CSM-FIR was a significant (*b* = -.10, *p* = .03); the impact of stress on anxiety was smaller for individuals who exhibited more electrodermal reactivity to the safety cue during acquisition. Notably, the value of anxiety change at the intercept represents anxiety change when all predictors are zero. Hence this represents change in anxiety when experiencing no stressors and no uplifts.

We plotted and probed the simple slope of stress on anxiety change at one SD above and below the group mean for CSM-FIR to better characterize how the fear learning construct related to within-person changes in anxiety. The form of the interaction is described in Figure 1 (upper left panel). Individuals lower in CSM-FIR (i.e., lower electrodermal reactivity to a safe cue during conditioning) correspondingly reported lower shifts in anxiety in safe moments during daily life (intercept = 1.63(.10), *p* < .001) and showed a significant increase in anxiety after a stressor (*Z* = 3.67, *p* < .001). However, individuals high in electrodermal reactivity to a safe cue during conditioning reported relatively higher shifts in anxiety during safe moments in daily life (i.e., no stressor, intercept = 2.04(.10), *p* < .001) and although they showed increased anxiety in the moments after a stressor, this was a non-significant change (*Z* = 1.16, *p* = .25); thus they remained elevated in anxiety across non-stressful and stressful moments in daily life.

Further, we plotted and probed the significant cross-level effect of gender on the stress on anxiety level 1 effect (*b* = -.46(.20), *p* = .02). As anticipated, and shown in Supplemental Figure 1, females generally had higher anxiety change across non-stressful moments (*b* = 1.95(.03), *p* < .001), and showed heightened anxiety change in response to stressful moments *Z* = 7.73, *p* < .001, relative to males (intercept, *b* = 1.72(.03), *p* < .001; slope, *Z* = 2.22, *p* = .03).

**CSM - LIR.** We next explored the two-level fixed effects model with CSM-LIR, which again indicated that higher STAI scores (*b* = .08(.02), *p* < .001) were associated with significantly higher momentary anxiety across the week (Table 2, third column from left). Next, we modelled a random slope for the within-cluster effect of stressor on pre- to post- stress anxiety, and the effect of stress on increased anxiety remained significant (*b* = .55(.10), *p* < .001). The slope variance was marginal/non-significant Z = 1.54 (Asparouhov, 2017), with the random slope specification showing improved model fit via -2LL difference test, reduced AIC, BIC, and level 1 residual variance.

We then modelled the cross-level interaction terms (Table 2, fourth column from left) to assess whether the predictive effect of stressor on anxiety change varied across individuals based on person-level predictors (CSM-LIR, gender). In this case, CSM-LIR was not a significant cross-level predictor of the impact of stress on anxiety change (*b* = -.08(.07), *p* = .24). Thus, electrodermal reactivity at late stages of CS- trials during acquisition did not predict differential anxiety change as a result of momentary stress.

However, gender did predict significant variance in the level 1 effect (*b* = -.41(.19), *p* = .03), and this effect was plotted and probed (Supplementary Figure 1). The pattern of findings for gender was in-line with expectations, in that females generally had higher anxiety change across non-stressful moments (*b* = 1.95(.06), *p* < .001), and showed heightened anxiety change in response to stressful moments *Z* = 5.93, *p* < .001, relative to males (intercept, *b* = 1.72(.06), *p* < .001; slope, *Z* = 2.28, *p* = .02).

**CSP – FIR.** As show in Table 1, two right columns, in the two-level fixed effects model, higher STAI scores were again associated with significantly higher momentary anxiety across the week (*b* = .08(.02), *p* < .001). Likewise, there was again a significant, positive main effect for CSP-FIR (*b* = .04(.02), *p* = .04).

Modelling a random slope for the within cluster effect of stressor on pre- to post-stress anxiety, the effect of stressor on increased anxiety remained significant (*b* = .55(.10), *p* < .001) and several indicators suggested the effect varied across individuals (i.e., slope variance was marginal to non-significant (t = 1.54), with a random slope specification that resulted in improved model fit as indicated by -2LL difference test, reduced AIC, BIC, and level 1 residual variance).

Estimating the random coefficients model with level 2 predictors (CSP-FIR, gender) of the random slope indicated both regression coefficients for the cross-level interactions were significant (Table 1, far right columns). The form of the CSP-FIR interaction (*b* = -.06*, p* = .04) is described in Figure 1, bottom left panel. Individuals at low levels of electrodermal reactivity to the CS+ correspondingly reported lower anxiety in safe moments during daily life and showed increases in anxiety in response to daily stressors. In contrast, individuals who acquired more electrodermal reactivity at the early stage of CS+ trials reported less change in anxiety from moments without relative to with a stressor, which was attributable to elevated anxiety across moments without a stressor which were similar (i.e., non-significant) across moments with stressors. Specifically, at low CSP-FIR scores (1 SD below the group mean), the intercept (intercept = 1.70(.09), *p* < .001) and slope were significant (*Z* = 2.67, *p* = .008). At high CSP-FIR scores (1 SD above the group mean), the intercept was relatively higher (intercept = 1.96(.10), *p* < .001) and the slope was non-significant (*Z* =.91, *p* = 0.36). Thus, greater electrodermal reactivity during early stages of CS+ trials during conditioning predicted greater anxiety in response to safe moments in daily life that did not discriminate in relation to stressful events.

Gender also predicted significant variance in the level 1 effect (*b* = -.43(.19), *p* = .02), and this effect was plotted and probed (Supplementary Figure 1). The pattern of findings for gender remained consistent. Females generally had higher anxiety change across non-stressful moments (*b* = 1.98(.02), *p* < .001), and showed heightened anxiety change in response to stressful moments *Z* = 6.25, *p* < .001, relative to males (intercept, *b* = 1.68(.02), *p* < .001; slope, *Z* = 2.24, *p* = .02).

**CSP – LIR.** Exploring the two-level fixed effect model, at level two, only STAI showed a significant, positive relation with momentary anxiety across the week (b = .08(.02), p < .001; Table 2 second column from right). We next modelled the random effect of stressor on anxiety change, and the positive effect of momentary stress on anxiety remained significant (*b* = .55 (.10), *p* < .001. Consistent with previous analyses, this effect appeared likely to vary across individuals, given a marginal slope variance (t = 1.54; Asparouhov, 2017) alongside improved model fit as indicated by ꭕ2 difference test, reduced AIC, BIC, and level 1 residual variance.

We then predicted variation in the effect of stressor on anxiety change via individual-level constructs of CSP-LIR, gender, and study type. As shown in Table 2 (right-most column), the cross-level interaction for CSP-LIR was not statistically significant after applying the Benjamini Hochberg false discovery rate (*b* = -.06(.03). Rather, only the cross-level interaction for gender was statistically significant (*b* = -.48(.19), *p* = .02). The nature of the interaction is characterized within Supplementary Figure 1 and is consistent with the gender pattern found previously. Females generally had higher anxiety change across non-stressful moments (*b* = 2.02(.03), *p* < .001), and showed heightened anxiety change in response to stressful moments *Z* = 7.38, *p* < .001, relative to to males (intercept, *b* = 1.65(.03), *p* < .001; slope, *Z* = 2.47, *p* = .014).

**Extinction**

**CSM – FIR.** As described in Table 3, in the level 1 and level 2 fixed effects model (third column from right), higher STAI-T scores were again associated with significantly higher reported anxiety across the week (b = .08(.02), *p* < .001). Next, a random slope was modelled for the within cluster effect of stressor on anxiety change. The positive effect of stressor on anxiety change again remained significant (*b* = .55(.10), *p* < .001), with indications that the effect varied across individuals. While the slope variance was estimated as marginal to non-significant (t = 1.6) (Asparouhov, 2017), the random slope specification resulted in improved model fit as indicated by -2LL difference test, reduced AIC, BIC, and level 1 residual variance.

We then estimated the random coefficients model with CSM-FIR during extinction, and gender as level 2 predictors of the random slope. As described in Table 3 (third column from right), only the cross-level interaction for gender was statistically significant (*b* = -.35(.17), *p* = .04). This interaction is characterized within Supplementary Figure 1. In line with patterns for gender across the study, females generally had higher anxiety change across non-stressful moments (*b* = 2.03(.14), *p* < .001), and showed relatively larger increases in anxiety in response to stressful moments *Z* = 1.70, *p* = .09, in comparison to males (intercept, *b* = 1.65(.14), *p* < .001; slope, *Z* = 1.03, *p* = .30).

**CSM – LIR.** As described in Table 4 (left), the level 1 and level 2 fixed effects model indicated that higher STAI scores were associated with significantly higher reported anxiety across the week (*b* = .08(.02) *p* < .001). We next modelled a random slope for the within cluster effect of stressor on anxiety change. Again, the positive effect of stressor on anxiety change remained significant (*b* = .55(.10), p < .001), with indications that the effect varied across individuals (slope variance t = 1.5 with random slope specification resulting in improved model fit per -2LL difference test, reduced AIC, BIC, and level 1 residual variance).

Estimating the random coefficients model with CSM-LIR during extinction and gender, only the CSM-LIR cross-level interaction was statistically significant after applying the Benjamini Hochberg false discovery rate (*b* = -.10(.05), *p* =.03). The top right panel of Figure 1 describes the interaction. As shown, individuals at low CSM-LIR levels during extinction showed significant increases in anxious reactivity in response to stressors. Whereas individuals higher on electrodermal reactivity to the CS- during extinction were generally the same across both non-stressful and stressful moments. Probing of the simple slopes indicate that for individuals at low levels of CSM-LIR during extinction (1 SD group mean), the intercept was slightly lower (intercept = 1.83(.09), p < .001) and the slope was steeper (*Z* = 4.88, *p* < .001) whereas for individuals scoring high (1 SD above the group mean) in CSM-LIR during extinction, the intercept was significant and slightly higher (intercept = 1.83(.08), *p* < .001) and the slope was not significant (*Z* = 1.73, *p* = .084). Thus, electrodermal reactivity during the later stages of responding to safe stimuli during extinction predicted greater anxiety in response to safe moments in daily life that did not discriminate in relation to stressful events.

**CSP – FIR.** In the level 1 and level 2 fixed effects model (Table 3, right), higher STAI scores were associated with significantly higher reported anxiety across the week (*b* = .08(.02), *p* <.001). Next, a random slope was modelled for the within cluster effect of stressor on anxiety change. The positive effect of stressor on subsequent anxiety again remained significant (*b* = .55(.10), *p* < .001 and likewise with indications that this effect varied across participants (t = 1.5), the random slope specification again indicated improved model fit as characterised by ꭕ2 difference test, reduced AIC, BIC, and level 1 residual variance. Estimating the random coefficients model with level 2 predictors (CSP-FIR, gender) of the random slope indicated neither of the regression coefficients for the cross-level interactions were significant (Table 3, far right column) after applying the Benjamini Hochberg false discovery rate, CSP-FIR *b =* -.06(.04), *p* = .08; gender *b* = -.38(.18).

**CSP – LIR.** In the two-level fixed effects model (Table 4, second column from right), higher STAI scores were again associated with significantly higher reported anxiety across the week (*b* = .08(.02), *p* < .001). Modelling a random slope for the within cluster effect of stressor on anxiety change, the effect of stressor on anxiety change was significant (*b* = .55(.10), *p* < .001). Again, several indicators indicated the effect varied across individuals (slope variance t = 1.49; random slope specification showed an improved model fit).

The two regression coefficients for the cross-level interactions were statistically significant (Table 4, right). First, examining the negative coefficient for CSP-LIR during extinction (*b* = -.16(.07), *p* = .02) again suggests that for participants with higher CSP-LIR during extinction, the impact of stressor on subsequent anxiety change was smaller than expected based on only the direct effect. Figure 1 (bottom right panel) describes the interaction. As shown, individuals high on CSP-LIR during extinction were generally similar following moments when no stressor occurred as when moments followed a stressor. Whereas individuals high on CSP-LIR remained high in anxiety across stressful moments, individuals low on CSP-LIR during extinction increased their anxious responses in relation to stressors. Probing of the simple slopes showed that for individuals at low levels of CSP-LIR during extinction, the intercept was relatively lower (intercept = 1.83(.09), *p* < .001) and the slope was significant (*Z* = 4.87, *p* < .001), whereas for individuals scoring high on CSP-LIR, the intercept was slightly higher and the slope was not significant (intercept = 1.83(.08), *p* < .001; slope *Z* = 1.73, *p* = .08).

Second, the cross-level interaction for gender was also statistically significant (*b* = -.36(.17), *p* = .04) and the form of the interaction is characterized within Supplementary Figure 1. In line with patterns for gender across the study, females generally had higher anxiety change across non-stressful moments (*b* = 2.03(.07), *p* < .001), and showed relatively larger increases in anxiety in response to stressful moments *Z* = 5.96, *p* < .001, in comparison to males (intercept, *b* = 1.65(.07), *p* < .001; slope, *Z* = 2.52, *p* = .02).

**Sensitivity Checks**

Finally, we ran sensitivity checks to our main analyses, substituting in the control variable of “Study” for Study Stimuli. Findings are reported in Supplementary Tables 2-5. First, no significant effects emerged for the study control variable. Further, for CSM-FIR and CSP-FIR findings were exactly the same. Likewise, for CSM-FIR and CSP-LIR findings were also the same. Whereas for CSP-LIR, sensitivity findings showed the cross-level interaction was significant with the FDR applied, in the main study findings the cross-level interaction was no longer significant once the FDR was applied. Further, for CSM-FIR, sensitivity findings showed the cross-level interaction was significant with the FDR applied, whereas in the main study findings, results were only trend (before applying FDR rate). Hence, across both groups of findings the exact same pattern of results emerged, with additional cross-level interactions meeting FDR significance within the Supplementary sensitivity checks. That said, our main study findings are considered conceptually more precise (controlling for stimuli used rather than study, the latter of which is arguably arbitrary), and were a better fit to the data. That is, in the main findings, AIC values were lower without exception, and BIC values were lower across 14 of 16 analyses, (all BIC values were lower in main study findings across Tables 1, 2, 4).

**References**

Asparouhov, T. (2018). Retrieved November 4, 2020 from variance of growth factors. <http://www.statmodel.com/discussion/messages/14/228.html?1395703447>

Modecki, K. L., Duvenage, M., Uink, B., Barber, B. L., & Donovan, C. L. (2022). Adolescents’ online coping: When less is more but none is worse. *Clinical Psychological Science, 10*(3), 467-481.

Modecki, K. L., Goldberg, R. E., Ehrenreich, S. E., Russell, M., & Bellmore, A. (2019). The practicalities and perils of ambulatory assessment's promise: Introduction to a special section. *Journal of Research on Adolescence*, *29*(3), 542-550.

Schneiders, J., Nicolson, N. A., Berkhof, J., Feron, F. J., Van Os, J., & Devries, M. W. (2006). Mood reactivity to daily negative events in early adolescence: relationship to risk for psychopathology. *Developmental Psychology, 42*(3), 543.

Silk, J. S., Steinberg, L., & Morris, A. S. (2003). Adolescents' emotion regulation in daily life: Links to depressive symptoms and problem behavior. *Child Development, 74*(6), 1869-1880.

Smyth, J. M., Wonderlich, S. A., Sliwinski, M. J., Crosby, R. D., Engel, S. G., Mitchell, J. E., & Calogero, R. M. (2009). Ecological momentary assessment of affect, stress, and binge‐purge behaviors: Day of week and time of day effects in the natural environment. *International Journal of Eating Disorders, 42*(5), 429-436.

Snijders, T. A., & Bosker, R. J. (1999). An introduction to basic and advanced multilevel modeling. London: Sage.

Uink, B., Modecki, K. L., Barber, B. L., & Correia, H. M. (2018). Socioeconomically disadvantaged adolescents with elevated externalizing symptoms show heightened emotion reactivity to daily stress: An experience sampling study. *Child Psychiatry & Human Development, 49*(5), 741-756.

Uink, B. N., Modecki, K. L., & Barber, B. L. (2017). Disadvantaged youth report less negative emotion to minor stressors when with peers: An experience sampling study. *International Journal of Behavioral Development*, *41*(1), 41-51.

van Roekel, E., Keijsers, L., & Chung, J. M. (2019). A review of current ambulatory assessment studies in adolescent samples and practical recommendations*. Journal of Research on Adolescence, 29*(3), 560-577.

|  | Shape CS’s & Scrape US  *N*=11 | Dogs CS’s & Growl + Scream US  *N*=40 |
| --- | --- | --- |
| Gender (male: female) | 6:5 | 9:31 |
| Age (*M, SD*) | 19.18 (2.18) | 18.80 (2.82) |
| STAI Trait (*M, SD*) | 46.75 (7.63) | 44.05 (11.45) |
| CA (% correct) | 100% | 100% |
| Sound intensity (*M, SD*) * | 4.27 (2.15) | 7.10 (1.74) |
| Sound unpleasantness (*M, SD*) * | 2.00 (1.90) | .48 (.96) |

Supplementary Table 1. Descriptive differences across stimuli.

**p* = < .001

Supplementary Table 2. First interval skin conductance responses to CSM and CSP during acquisition and anxiety models, sensitivity check.

|  | b (SE)  95% CI | b (SE)  95% CI | b (SE)  95% CI | b (SE)  95% CI | b (SE)  95% CI | b (SE)  95% CI |
| --- | --- | --- | --- | --- | --- | --- |
|  | Null model | Level 1 model | Two level model  CSM-FIR | **Cross level interactions**  **CSM-FIR** | Two level model  CSP-FIR | **Cross level interactions CSP-FIR** |
| **Intercept** | 1.86(.12), p < .001  [1.63-2.09] | 1.85(.11), p < .001  [1.63-2.08] | 1.83(.09), p < 001*  [1.66-2.01] | 1.83(.09), p <.001*  [1.66-2.01] | 1.83(.09), p < .001*  [1.65-2.01] | 1.83(.09), p < .001*  [1.65-2.01] |
| **Within** |  |  |  |  |  |  |
| Time |  | -.02(.01), p =.17  [-.04 ─ .01] | -.01(.01), p = .34  [-.04 ─ .01] | -.01(.01), p = .25  [-.04 ─ .01] | -.01(.01), p = .34  [-.04 ─ .01] | -.02(.01), p = .25  [-.04 ─ .01] |
| Uplift |  | -.14(.06), p = .02  [-.26 ─ -.02] | -.14(.07), p = .04  [-.27 ─ .01] | -.13(.06), p = .03  [-.25 ─ -.01] | -.14(.07), p = .04*  [-.27 ─ -.01] | -.13(.06), p = .03*  [-.25 ─ .01] |
| Stressor |  | .55(.09), p < .001  [.37 ─ .73] | .53(.10), p < .001*  [.34 ─ .71] | .54(.09), p < .001*  [.36 ─ .72] | .53(.01), p < .001*  [.34 ─ .71] | .55(.09), p < .001*  [.37─ .73] |
| Anx Lagged |  | .04(.02), p =.12  [-.01 ─ .09] | .04(.03), p = .10  [-.01 ─ .09] | .05(.03), p = .07  [-.01 ─ .10] | -.04(.03), p = .10  [-.01 ─ .09] | .05(.03), p = .06  [-.01 ─ .10] |
| Variance Within | 1.40(.18), p < .001  [1.05 ─ 1.75] | .67(.08), p < .001  [.52 ─ .82] | .66(.08), p < .001*  [.50 ─ .81] | .63(.08), p < .001*  [.48 ─ .79] | .66(.08), p < .001*  [.50 ─ .81] | .64(.08), p < .001*  [.48 ─ .79] |
| **Between** |  |  |  |  |  |  |
| Study |  |  | -.11(.14), p = .40  [-.38 ─ .15] | -.11(.14), p = .41  [-.38 ─ .16] | -.11(.14), p = .43  [-.38 ─ .16] | -.11(.14), p = .45  [-.38 ─ .17] |
| Start Day |  |  | -.18(.08), p = .02*  [-.33 ─ .03] | -.18(.08), p = .02*  [-.33 ─ -.02] | -.20(.09), p = .02*  [-.36 ─ -.03] | -.20(.09), p = .02*  [-.37 ─ -.03] |
| STAI-T |  |  | .08(.02), p < .001*  [.05 ─ .11] | .08(.02), p < .001*  [.05 ─ .11] | .08(.02), p < .001*  [.05 ─ .11] | .08(.02), p < .001*  [.05 ─ .11] |
| Gender |  |  | -.32(.22), p = .15  [-.76 ─ .12] | -.32(.23), p = .17  [-.77 ─ .12] | -.39(.25), p = .12  [-.87 ─ .10] | -.39(.25), p = .12  [-.89 ─ .10] |
| CSM/P-FIR |  |  | .09(.03), p = .01*  [.02 ─ .15] | .09(.03), p = .01*  [.02 ─ .15] | .04(.02), p =.07  [-.01 ─ .08] | .04(.02), p = .07  [-.01 ─ .09] |
| Slope Study |  |  |  | .12(.08), p = .12  [-.03 - .27] |  | .17(.10), p = .07  [-.02 - .36] |
| Slope Gender |  |  |  | -.37(.20), p = .06  [-.76 ─ .01] |  | -.31(.20), p = .12  [-.69 ─ .08] |
| Slope CSM/P-FIR |  |  |  | -.09(.04), p = .02*  [-.17 ─ .01] |  | -.06(.03), p = .03*  [-.12 ─ -.01] |
| Slope Variance |  |  |  | .17(.12), p = .18  [-.08 ─ .41] |  | .16(.13), p = .23  [-.10 ─ .41] |
| Variance Between |  | .67(.16), p <.001  [.35 ─ .98] | .33(.07), p < .001*  [.20 ─ .47] | .33(.07), p < .001*  [.20 ─ .47] | .35(.07), p < .001*  [.21 ─ .49] | .35(.07), p < .001*  [.21 ─ .49] |
| **AIC** | 6205.23 | 4342.64 | 4408.90 | 4392.51 | 4411.74 | 4395.94 |
| **BIC** | 6216.39 | 4380.70 | 4484.51 | 4495.12 | 4487.35 | 4498.55 |

*Note.* Stressor = 0 = no bad event since last messaged, 1 = bad event since last messaged; Uplift = 0 = no uplift since last messaged, 1 = uplifting event since last messaged; Start day = Monday = 1 to Sunday = 7; STAI-T = Trait anxiety score ÷ 2. Gender = 0 = female, 1 = male. Slope Coefficients represent the cross-level interactions (e.g., level-2 moderator of the level-1 anxiety on stressor regression). Two-level and cross-level model coefficients which met FDR-corrected significance level marked with an asterisk.

Supplementary Table 3. Last interval skin conductance responses to CSM and CSP during acquisition and anxiety models, sensitivity check.

|  | b (SE)  95% CI | b (SE)  95% CI | b (SE)  95% CI | b (SE)  95% CI | b (SE)  95% CI | b (SE)  95% CI |
| --- | --- | --- | --- | --- | --- | --- |
|  | Null model | Level 1 model | Two level model  CSM-LIR | **Cross level interactions**  **CSM-LIR** | Two level model  CSP-LIR | **Cross level interactions CSP-LIR** |
| **Intercept** | 1.86(.12), p < .001  [1.63-2.09] | 1.85(.11), p < .001  [1.63-2.08] | 1.83(.09), p < .001*  [1.65 – 2.02] | 1.83(.09), p < .001*  [1.65-2.02] | 1.83(.09), p < .001*  [1.65-2.02] | 1.83(.09), p < .001*  [1.65-2.02] |
| **Within** |  |  |  |  |  |  |
| Time |  | -.02(.01), p =.17  [-.04 ─ .01] | -.01(.01), p = .34  [-.04 ─ .01] | -.01(.01), p = .26  [-.04 ─ .01] | -.01(.01), p = .34  [-.04 ─ .01] | -.01(.01), p = .26  [-.04 ─ .01] |
| Uplift |  | -.14(.06), p = .02  [-.26 ─ -.02] | -.14(.07), p = .04  [-.27 ─ -.01] | -.14(.06), p = .03  [-.26 ─ -.01] | -.14(.07), p = .04*  [-.27 ─ -.01] | -.13(.06), p = .03*  [-.25 ─ -.01] |
| Stressor |  | .55(.09), p < .001  [.37 ─ .73] | .53(.10), p < .001*  [.34 ─ .71] | .55(.10), p < .001*  [.36 ─ .74] | .53(.10), p <.001*  [.34 ─ .71] | .56(.09), p <.001*  [.38 ─ .74] |
| Anx Lagged |  | .04(.02), p =.12  [-.01 ─ .09] | .04(.03), p = .10  [-.01 ─ .09] | .05(.03), p = .08  [-.01 ─ .10] | .04(.03), p = .10  [-.01 ─ .09] | .05(.03), p = .06  [-.01 ─ .09] |
| Variance Within | 1.40(.18), p < .001  [1.05 ─ 1.75] | .67(.08), p < .001  [.52 ─ .82] | .66(.08), p < .001*  [.50 ─ .81] | .64(.08), p < .001*  [.48 ─ .79] | .66(.08), p < .001*  [.50 ─ .81] | .64(.08), p < .001*  [.48 ─ .79] |
| **Between** |  |  |  |  |  |  |
| Study |  |  | -.10(.14), p = .47  [-.37 ─ .17] | -.10(.14), p = .49  [-.37 ─ .18] | -.10(.13), p = .45  [-.36 ─ .16] | -.10(.13), p = .46  [-.36 ─ .16] |
| Start Day |  |  | -.16(.08), p = .06  [-.31 ─ .01] | -.16(.08), p = .05  [-.32 ─ .01] | -.19(.09), p = .03  [-.36 ─ -.02] | -.19(.09), p = .03  [-.36 ─ -.02] |
| STAI-T |  |  | .08(.02), p < .001*  [.05 ─ .12] | .08(.02), p < .001*  [.05 ─ .12] | .08(.02), p < .001*  [.04 ─ .11] | .08(.02), p < .001*  [.04 ─ .11] |
| Gender |  |  | -.31(.14), p = .47  [-.77 ─ .16] | -.31(.24), p = .20  [-.79 ─ .16] | -.45(.24), p = .06  [-.92 ─ .02] | -.45(.24), p = .06  [-.93 ─ .02] |
| CSM/P-LIR |  |  | .11(.05), p = .05  [.01 ─ .21] | .11(.05), p = .05  [.01 ─ .21] | -.01(.03), p = .89  [-.05 ─ .06] | -.01(.03), p = .88  [-.05 ─ .06] |
| Slope Study |  |  |  | .10(.09), p = .27  [-.08 - .28] |  | .20(.10), p = .04  [.01 - .38] |
| Slope Gender |  |  |  | -.32(.20), p = .11  [-.72 ─ .07] |  | -.31(.20), p = .11  [-.69 ─ .08] |
| Slope CSM/P-LIR |  |  |  | -.07(.07), p = .31  [-.21 ─ .07] |  | -.07(.03), p = .01*  [-.12 ─ -.02] |
| Slope Variance |  |  |  | .20(.14), p = .16  [-.08 ─ .47] |  | .14(.12), p = .26  [-.10 ─ .38] |
| Variance Between |  | .67(.16), p <.001  [.35 ─ .98] | .34(.07), p <.001*  [.22 ─ .47] | .34(.06), p < .001*  [.22 ─ .47] | .37(.08), p < .001*  [.22 ─ .52] | .37(.08), p < .001*  [.22 ─ .52] |
| **AIC** | 6205.23 | 4342.64 | 4411.16 | 4398.79 | 4413.87 | 4396.21 |
| **BIC** | 6216.39 | 4380.70 | 4486.76 | 4501.40 | 4489.48 | 4498.83 |

*Note.* Stressor = 0 = no bad event since last messaged, 1 = bad event since last messaged; Uplift = 0 = no uplift since last messaged, 1 = uplifting event since last messaged; Start day = Monday = 1 to Sunday = 7; STAI-T = Trait anxiety score ÷ 2. Gender = 0 = female, 1 = male. Slope Coefficients represent the cross-level interactions (e.g., level-2 moderator of the level-1 anxiety on stressor regression). Two-level and cross-level model coefficients which met FDR-corrected significance level marked with an asterisk.

Supplemental Table 4. First interval skin conductance responses to CSM and CSP during extinction and anxiety models, sensitivity check.

|  | b (SE)  95% CI | b (SE)  95% CI | b (SE)  95% CI | b (SE)  95% CI | b (SE)  95% CI | b (SE)  95% CI |
| --- | --- | --- | --- | --- | --- | --- |
|  | Null model | Level 1 model | Two level model  CSM-FIR | **Cross level interactions**  **CSM-FIR** | Two level model  CSP-FIR | **Cross level interactions CSP-FIR** |
| **Intercept** | 1.86(.12), p < .001  [1.63-2.09] | 1.85(.11), p < .001  [1.63-2.08] | 1.83(.09), p < .001*  [1.65-2.02] | 1.83(.09), p < .001*  [1.65-2.02] | 1.83(.09), p < .001*  [1.65-2.01] | 1.83(.09), p < .001*  [1.65-2.01] |
| **Within** |  |  |  |  |  |  |
| Time |  | -.02(.01), p =.17  [-.04 ─ .01] | -.01(.01), p =.34  [-.04 ─ .01] | -.01(.01), p =.27  [-.04 ─ .01] | -.01(.01), p = .34  [-.04 ─ .01] | -.01(.01), p = .26  [-.04 ─ .01] |
| Uplift |  | -.14(.06), p = .02  [-.26 ─ -.02] | -.14(.07), p = .04  [-.27 ─ -.01] | -.13(.06), p = .03  [-.25 ─ -.01] | -.14(.07), p = .04*  [-.27 ─ -.01] | -.13(.06) p = .03*  [-.26 ─ -.01] |
| Stressor |  | .55(.09), p < .001  [.37 ─ .73] | .53(.10), p < .001*  [.34 ─ .71] | .55(.09), p < .001*  [.37 ─ .73] | .53(.10), p < .001*  [.34 ─ .71] | .55(.10), p < .001*  [.37 ─ .74] |
| Anx Lagged |  | .04(.02), p =.12  [-.01 ─ .09] | .04(.03), p = .10  [-.01 ─ .09] | .05(.03), p = .07  [-.01 ─ .10] | .04(.03), p = .10  [-.01 ─ .09] | .05(.03), p = .07  [-.01 ─ .10] |
| Variance Within | 1.40(.18), p < .001  [1.05 ─ 1.75] | .67(.08), p < .001  [.52 ─ .82] | .66(.08), p < .001*  [.50 ─ .81] | .64(.08), p < .001*  [.48 ─ .79] | .66(.08), p < .001*  [.50 ─ .81] | .64(.08), p < .001*  [.48 ─ .79] |
| **Between** |  |  |  |  |  |  |
| Study |  |  | -.11(.13), p = .40  [-.38 ─ .15] | -.11(.14), p = .41  [-.38 ─ .15] | -.12(.13), p = .36  [-.39 ─ .14] | -.12(.13), p = .37  [-.38 ─ .14] |
| Start Day |  |  | -.19(.09), p = .03*  [-.36 ─ -.02] | -.19(.09), p = .02*  [-.36 ─ -.03] | -.20(.08), p = .02*  [-.36 ─ -.03] | -.20(.08), p = .02*  [-.36 ─ -.03] |
| STAI-T |  |  | .08(.02), p < .001*  [.04 ─ .11] | .08(.02), p < .001*  [.04 ─ .11] | .08(.02), p < .001*  [.05 ─ .11] | .08(.02), p < .001*  [.05 ─ .12] |
| Gender |  |  | -.46(.24), p = .06  [-.93 ─ .01] | -.47(.25), p = .05  [-.52 ─ .16] | -.42(.23), p = .07  [-.87 ─ .04] | -.42(.24), p = .08  [-.88 ─ .04] |
| CSM/P-FIR |  |  | -.04(.07), p = .57  [-.09 ─ .17] | .04(.07), p = .58  [-.09 ─ .17] | .10(.04), p = .01*  [.02 ─ .17] | .10(.04), p = .01  [.02 ─ .17] |
| Slope Study |  |  |  | .21(.11), p = .05  [.01 - .42] |  | .17(.10), p = .08  [-.02 - .37] |
| Slope Gender |  |  |  | -.18(.17), p = .31  [-.52 ─ .16] |  | -.26(.18), p = .16  [-.61 ─ .10] |
| Slope CSM/P-FIR |  |  |  | -.16(.07), p = .02*  [-.31 ─ -.02] |  | -.08(.04), p = .06  [-.16 ─ .01] |
| Slope Variance |  |  |  | .16(.12), p = .18  [-.07 ─.40] |  | .18(.14), p =.18  [-.09 ─ .45] |
| Variance Between |  | .67(.16), p <.001  [.35 ─ .98] | .37(.07), p < .001*  [.22 ─ .51] | .37(.08), p <.001*  [.22 ─ .51] | .33(.07), p < .001*  [.20 ─ .46] | .33(.07), p < .001*  [.20 ─ .47] |
| **AIC** | 6205.23 | 4342.64 | 4413.60 | 4396.82 | 4409.85 | 4396.14 |
| **BIC** | 6216.39 | 4380.70 | 4489.21 | 4499.43 | 4485.46 | 4498.75 |

*Note.* Stressor = 0 = no bad event since last messaged, 1 = bad event since last messaged; Uplift = 0 = no uplift since last messaged, 1 = uplifting event since last messaged; Start day = Monday = 1 to Sunday = 7; STAI-T = Trait anxiety score ÷ 2. Gender = 0 = female, 1 = male. Slope Coefficients represent the cross-level interactions (e.g., level-2 moderator of the level-1 anxiety on stressor regression). Two-level and cross-level model coefficients which met FDR-corrected significance level marked with an asterisk.

Supplementary Table 5. Last interval skin conductance responses to CSM and CSP during extinction and anxiety models, sensitivity check.

|  | b (SE)  95% CI | b (SE)  95% CI | b (SE)  95% CI | b (SE)  95% CI | b (SE)  95% CI | b (SE)  95% CI |
| --- | --- | --- | --- | --- | --- | --- |
|  | Null model | Level 1 model | Two level model  CSM-LIR | **Cross level interactions**  **CSM-LIR** | Two level model  CSP-LIR | **Cross level interactions CSP-LIR** |
| **Intercept** | 1.86(.12), p < .001  [1.63-2.09] | 1.85(.11), p < .001  [1.63-2.08] | 1.83(.09), p < .001*  [1.65-2.02] | 1.83(.09), p < .001*  [1.65-2.02] | 1.83(.09), p < .001*  [1.65-2.02] | 1.83(.09), p < .001*  [1.65-2.02] |
| **Within** |  |  |  |  |  |  |
| Time |  | -.02(.01), p =.17  [-.04 ─ .01] | -.01(.01), p = .34  [-.04 ─ .01] | -.01(.01), p = .28  [-.04 ─ .01] | -.01(.01), p = .34  [-.04 ─ .01] | -.01(.01), p = .27  [-.04 ─ .01] |
| Uplift |  | -.14(.06), p = .02  [-.26 ─ -.02] | -.14(.07), p = .04  [-.27 ─ .01] | -.14(.06), p = .03*  [-.26 ─ -.02] | -.14(.07), p = .04*  [-.27 ─ -.01] | -.14(.06), p = .02*  [-.26 ─ -.02] |
| Stressor |  | .55(.09), p < .001  [.37 ─ .73] | .53(.10), p < .001*  [.34 ─ .71] | .56(.10), p < .001*  [.38 ─ .75] | .53(.10), p < .001*  [.34 ─ .71] | .56(.09), p < .001*  [.38 ─ .74] |
| Anx Lagged |  | .04(.02), p =.12  [-.01 ─ .09] | .04(.03), p = .10  [-.01 ─ .09] | .05(.03), p = .07  [-.01 ─ .10] | .04(.03), p = .10  [-.01 ─ .09] | .05(.03), p = .08  [-.01 ─ .10] |
| Variance Within | 1.40(.18), p < .001  [1.05 ─ 1.75] | .67(.08), p < .001  [.52 ─ .82] | .66(.08), p < .001*  [.50 ─ .81] | .64(.08), p < .001*  [.48 ─ .79] | .66(.08), p < .001*  [.50 ─ .81] | .64(.08), p < .001*  [.48 ─ .79] |
| **Between** |  |  |  |  |  |  |
| Study |  |  | -.10(.14), p = .46  [-.37 ─ .17] | -.09(.14), p = .49  [-.36 ─ .18] | -.10(.13), p = .44  [-.37 ─ .16] | -.10(.14), p = .47  [-.36 ─ .17] |
| Start Day |  |  | -.19(.08), p = .03  [-.35 ─ -.02] | -.19(.08), p = .02*  [-.36 ─ -.03] | -.19(.09), p = .03*  [-.35 ─ -.02] | -.19(.09), p = .03*  [-.36 ─ -.02] |
| STAI-T |  |  | .08(.02), p < .001*  [.04 ─ .11] | .07(.02), p < .001*  [.04 ─ .11] | .08(.02), p < .001*  [.04 ─ .11] | .08(.02), p < .001*  [.04 ─ .11] |
| Gender |  |  | -.45(.24), p = .06  [-.92 ─ .02] | -.46(.25), p = .06  [-.95 ─ .02] | -.45(.24), p = .06  [-.92 ─ .02] | -.46(.25), p = .06  [.04 ─ .11] |
| CSM/P-LIR |  |  | -.01(.06), p = .96  [-.11 ─ .11] | -.01(.06), p = .93  [-.11 ─ .10] | .01(.07), p = .86  [-.13 ─ .15] | .01(.07), p = .86  [-.13 ─ .15] |
| Slope Study |  |  |  | .17(.10), p = .09  [-.03 - .37] |  | .14(.10), p = .15  [-.05 - .34] |
| Slope Gender |  |  |  | -.22(.18), p = .22  [-.58 ─ -.13] |  | -.25(.18), p = .17  [-.61 ─ .11] |
| Slope CSM/P-LIR |  |  |  | -.12(.05), p = .01*  [-.21 ─ -.03] |  | -.16(.07), p = .02*  [-.29 ─ -.03] |
| Slope Variance |  |  |  | .17(.13), p = .19  [-.09 ─ .43] |  | .17(.13), p = .21  [-.09 ─ .43] |
| Variance Between |  | .67(.16), p <.001  [.35 ─ .98] | .37(.08), p < .001*  [.22 ─ .52] | .37(.08), p < .001*  [.22 ─ .52] | .37(.08), p < .001*  [.22 ─ .52] | .37(.08), p < .001*  [.22 ─ .52] |
| **AIC** | 6205.23 | 4342.64 | 4413.88 | 4398.55 | 4413.86 | 4398.16 |
| **BIC** | 6216.39 | 4380.70 | 4489.49 | 4501.16 | 4489.47 | 4500.77 |

*Note.* Stressor = 0 = no bad event since last messaged, 1 = bad event since last messaged; Uplift = 0 = no uplift since last messaged, 1 = uplifting event since last messaged; Start day = Monday = 1 to Sunday = 7; STAI-T = Trait anxiety score ÷ 2. Gender = 0 = female, 1 = male. Slope Coefficients represent the cross-level interactions (e.g., level-2 moderator of the level-1 anxiety on stressor regression). Two-level and cross-level model coefficients which met FDR-corrected significance level marked with an asterisk.

Supplemental Figure 1. Plots of significant cross-level interactions of gender on the stress-emotion regression term, within main study findings.

**Acquisition**

Supplemental Figure 1 Continued. Plots of significant cross-level interactions of gender on the stress-emotion regression term, within main study findings.

**Extinction**

**Extinction**
